# Molecular subtypes based on centrosome-related genes can predict prognosis and therapeutic responsiveness in patients with low-grade gliomas

**DOI:** 10.3389/fonc.2023.1157115

**Published:** 2023-03-27

**Authors:** Ganghua Zhang, Panpan Tai, Jianing Fang, Aiyan Chen, Xinyu Chen, Ke Cao

**Affiliations:** Department of Oncology, Third Xiangya Hospital of Central South University, Changsha, China

**Keywords:** centrosome, LGG, prognosis, immunotherapy, temozolomide

## Abstract

**Background:**

Abnormalities in centrosome regulatory genes can induce chromosome instability, cell differentiation errors, and tumorigenesis. However, a limited number of comprehensive analyses of centrosome-related genes have been performed in low-grade gliomas (LGG).

**Methods:**

LGG data were extracted from The Cancer Genome Atlas (TCGA) and Chinese Glioma Genome Atlas (CGGA) databases. The ConsensusClusterPlus” R package was used for unsupervised clustering. We constructed a centrosome-related genes (CRGs) signature using a random forest model, lasso Cox model, and multivariate Cox model, and quantified the centrosome-related risk score (centS). The prognostic prediction efficacy of centS was evaluated using a Receiver Operating Characteristic (ROC) curve. Immune cell infiltration and genomic mutational landscapes were evaluated using the ESTIMATE algorithm, single-sample Gene Set Enrichment Analysis (ssGSEA) algorithm, and “maftools” R package, respectively. Differences in clinical features, isocitrate dehydrogenase (IDH) mutation, 1p19q codeletion, O^6^-methylguanine-DNA methyltransferase promoter (MGMTp) methylation, and response to antitumor therapy between the high- and low-centS groups were explored. “pRRophetic” R packages were used for temozolomide (TMZ) sensitivity analysis. qRT-PCR verified the differential expression of the centrosomal gene team, the core of which is CEP135, between LGG cells and normal cells.

**Results:**

Two distinct CRG-based clusters were identified using consensus unsupervised clustering analysis. The prognosis, biological characteristics, and immune cell infiltration of the two clusters differed significantly. A well-performing centS signature was developed to predict the prognosis of patients with LGG based on 12 potential CRGs. We found that patients in the high-centS group showed poorer prognosis and lower proportion of IDH mutation and 1p19q codeletion compared to those in the low-centS group. Furthermore, patients in the high-centS group showed higher sensitivity to TMZ, higher tumor mutation burden, and immune cell infiltration. Finally, we identified a centrosomal gene team whose core was CEP135, and verified their differential expression between LGG cells and normal glial cells.

**Conclusion:**

Our findings reveal a novel centrosome-related signature for predicting the prognosis and therapeutic responsiveness of patients with LGG. This may be helpful for the accurate clinical treatment of LGG.

## Introduction

Gliomas are the most common primary malignant tumors of the central nervous system (1). According to the histological differences in glioma, the World Health Organization (WHO) divides glioma into four grades: grade I, II, III, and IV. Grade II and III tumors are low-grade gliomas (LGG), including astrocytomas, oligodendrogliomas, oligoastrocytomas, and anaplastic astrocytomas, and have better prognosis compared to glioblastoma (GBM, WHO Grade IV) (2). Surgery is the preferred treatment for LGG, but it is often impossible to completely remove LGGs because of their diffuse invasive nature or proximity to important structures. Currently, the dominant LGG treatment is surgery followed by radiotherapy. Chemotherapy, mainly including procarbazine, lomustine, and vincristine (PCV) regimens, as well as TMZ monotherapy, is a promising alternative therapy. National Comprehensive Cancer Network guidelines indicate that TMZ monotherapy remains the most important chemotherapy regimen for patients with LGG (3). The EORTC 22033-26033 study showed no significant difference in progression-free survival (PFS) in patients with LGG treated with radiotherapy or TMZ alone (4). IDH, TP53, alpha-thalassemia/mental retardation, X-linked (ATRX) mutations, MGMTp methylation, and 1p/19q codeletion have been considered clinically meaningful markers of LGG (5–8), and especially IDH mutation, 1p/19q codeletion, and MGMTp methylation status are closely related to diagnosis and prognosis, which are crucial for postsurgical treatment such as adjuvant chemotherapy and adjuvant radiotherapy (9). The median survival time for LGG is 5 to 10 years with the current standard treatment (10). However, due to LGG’s high intratumoral heterogeneity and diverse clinical behavior (11), some patients with LGG quickly advance to GBM (12). Therefore, there is an urgent need to identify the underlying molecular mechanisms and construct an effective molecular classification model to evaluate the prognosis and guide the individualized treatment of patients with LGG.

As the major microtubule-organizing center, the centrosome, composed of two centrioles and pericentriolar material, plays a crucial role in various cellular processes, including chromosome segregation, spindle formation, and cell division (13, 14). Dysfunction of core centrosomal or centrosome/centriole-associated proteins is connected to cell-cycle misregulation, chromosome segregation error, and cancer (15). Centrosome abnormalities have been reported in multiple cancer types, such as ovarian, breast, lung cancers, and multiple myeloma (16–19). Gliomas may arise from neural stem cells or progenitor cells and the chromosome segregation mechanism plays a vital role in the regulation of self-renewal and differentiation of these cells. Abnormalities in centrosome- and microtubule function-related genes can lead to serious errors in the differentiation of neural stem and progenitor cells (20). A recent study showed that the centromere protein J (CENPJ) is overexpressed in human GBM and that it promotes GBM progression, but there are few studies on the effects of aberrant centrosome-associated proteins on LGG. Hence, we selected 13 crucial proteins, polo-like kinase 4 (PLK4), SCL/TAL interrupting locus (STIL), spindle assembly abnormal protein 6 homolog (SAS6), spindle and centriole associated protein 1 (SPICE1), centrosomal protein 295 (CEP295), centrosomal protein POC5, CEP152, CENPJ/centrosomal P4.1-associated protein (CPAP), rotatin (RTTN), CEP135, CEP63, POC1 centriolar protein B (POC1B), and CEP120, that are closely related to centrosome/centriole activity from the literature to comprehensively explore the effect of centrosomal proteins on LGG treatment, recurrence progression, and prognosis. We named the genes that encode these proteins as centrosome-related genes (CRGs). PLK4, a master regulator of centrioles, is recruited to the mother centriole by CEP152 and CEP192 and is activated by binding to STIL. SAS6 is then recruited, which in turn starts the construction of the cartwheel, a structural platform for procentriole formation (21). CEP63 forms a complex with CEP152, which is essential for maintaining a normal centrosome number (22). The creation and maintenance of the procentriole microtubule wall is aided by CENPJ/CPAP, which works in conjunction with its binding partners CEP135, CEP120, and SPICE1 (21). RTTN interacts with STIL and is recruited close to the procentriole during the S-phase, acting downstream of the STIL-mediated centriole assembly (23). RTTN is necessary for the proper loading of centromeric proteins POC5, POC1B, and CETN1 to the distal centriole at a later stage, as well as for the appropriate loading of CEP295 to the proximal centrioles (23, 24).

In this study, we conducted twice subtype clustering based on key centrosome related genes on LGG’s samples and found significant clinicopathological, biological, and prognostic differences among the subtypes. Furthermore, we established a prediction signature, centrosome score (centS), based on 12 potential centrosome genes and divided the samples into high- and low-centS groups according to the centS. Our analysis revealed that the low-score group had better prognosis, higher percentage of IDH mutation, MGMTp methylation, and1p/19q codeletion, and preferable antitumor therapy outcome, but lower tumor mutation burden (TMB), immune infiltration, and TMZ sensitivity. Finally, we explored the differential expression of the key gene CEP135 and three potential CRGs that are strongly associated with key genes between LGG and normal glial cells.

## Materials and methods

### Data collection and processing

We obtained the RNA-seq data in transcripts per kilobase million (TPM) format from The Cancer Genome Atlas (TCGA) and Genome Tissue Expression (GTEx), which were processed uniformly by the Toil process in UCSC Xena (https://xenabrowser.net/datapages/) for differential gene expression (DGE) analysis (25). TCGA-LGG data (515 samples), and normal tissue data from GTEx (1152 samples) were extracted. RNA sequencing data (fragments per kilobase million, FPKM values) gene expression and clinical data of LGG were obtained from TCGA. The FPKM values were then converted to TPM for further analysis, and samples without complete survival data were excluded. Finally, we obtained a TCGA-LGG cohort containing 506 samples. The baseline data for all TCGA-LGGs are presented in **Table 1**. LGG samples from the Chinese Glioma Genome Atlas (CGGA, http://www.cgga.org.cn/) were screened, and samples without survival data were excluded. A total of 592 samples were selected, as shown in **Table 2**.

**Table 1:** Baseline Data Sheet for the Cohort of TCGA-LGG.

Characteristic	Levels	N (%)
Age	>45 years old	204 (39.6%)
	≤45 years old	311 (60.4%)
Gender	Male	285 (55.3%)
	Female	230 (44.7%)
Grade	G2	249 (48.4%)
	G3	266 (51.6%)
histological_type	Oligodendroglioma	191 (37.1%)
	Oligoastrocytoma	130 (25.2%)
	Astrocytoma	194 (37.7%)

**Table 2:** Baseline Data Sheet for the Cohort of CGGA-LGG.

Characteristic	Levels	N (%)
Age	>45 years old	160 (27.0%)
	≤45 years old	432 (73.0%)
Gender	Male	341 (57.6%)
	Female	251 (42.4%)
Grade	WHO II	270 (45.6%)
	WHO III	322 (54.4%)
histological_type	A	160 (27.0%)
	O	106 (17.9%)
	OA	9 (1.5%)
	AA	206 (34.8%)
	AO	91 (15.4%)
	AOA	20 (3.4%)
IDH_mutation	Wildtype	138 (25.0%)
	Mutant	415 (75.0%)
1p19q_codeletion	Non-codel	372 (67.4%)
	Codel	180 (32.6%)
MGMTp_methylation	Un-methylated	200 (41.2%)
	Methylated	285 (58.8%)

### Genetics, expression, and survival analysis of CRGs in LGG

Thirteen important CRGs from the published articles were screened. The Gene Set Cancer Analysis (GSCA) database (http://bioinfo.life.hust.edu.cn/GSCA/#/) was used to analyze the single nucleotide variation (SNV) and copy number variation (CNV) of CRGs (26). CNV data were downloaded from https://xena.ucsc.edu/. The “limma” R package was used to analyze the differentially expressed genes (DEGs). The Kaplan-Meier (K-M) survival curve showed the effect of CRGs on LGG prognosis. The “igraph” R package was used to draw a prognostic correlation network based on correlation analysis and univariate Cox regression analysis.

### Unsupervised clustering based on CRGs

All samples were clustered to different subtypes using the “ConsensusClusterPlus” R package according to the expression of the 13 CRGs (27). Survival analysis was performed to compare the differences in overall survival (OS) between the two subtypes, and DGE analysis was used to examine the expression of the 13 CRGs among different subtypes. The “pheatmap” R package was utilized to draw a cluster heat map to show the distribution of the expression of the 13 CRGs and clinicopathological features in different subtypes.

### Gene set variation analysis

The gene set data from the Kyoto Encyclopedia of Genes and Genomes (KEGG), Reactome, and HALLMARK pathways were downloaded from the Molecular Signatures Database (MsigDB,http://software.broadinstitute.org/gsea/msigdb/). The following gene sets served as references: “c2.cp.kegg.v7.5.1. symbols.gmt”, “c2.cp.reactome.v7.5.1. symbols.gmt,” and “h.all.v7.5.1. symbols.gmt” (28). Then, the “GSVA” R package was used to perform GSVA to explore the potential pathways whose activities vary in different subtypes.

### Identification of DEGs and enrichment analysis

The “limma” R package was used to screen DEGs among different subtypes with |logFoldChange|>1 and p<0.05. KEGG and Gene Ontology (GO) functional enrichment analyses were performed using the “clusterProfiler” R package (29). An adjusted p value of <0.05 was considered statistically significant.

### Unsupervised clustering based on centrosome subtype-related genes

Univariate Cox regression analysis was used to screen prognostic DEGs (p<0.05). Subsequently, using the same specific clustering parameters, unsupervised clustering classification was performed based on the prognostic DEGs, and the samples were divided into different gene subtypes. We then performed survival analysis to compare the differences in OS among different gene subtypes, implemented DGEs analysis to compare CRGs expression between different gene subtypes, and drew a cluster heat map to illustrate the connection between DEGs expression, clinicopathological characteristics, and gene subtypes.

### Construction and evaluation of the centrosome signature

Least absolute shrinkage and selection operator (Lasso) Cox regression analysis and the random forest algorithm were used to reduce dimensionality and screen feature genes based on the prognostic DEGs. The “glmnet” R package was used to execute Lasso Cox regression analysis. Random forest is an integrated algorithm based on decision tree classifiers. Bootstrap self-help method puts back and extracts k sample sets to form k decision trees. With a default of 100 iterations and 500 trees built, the model is robust enough. The random forest is based on Gini coefficient to select gene features. We used the “randomForest” R package to screen genes with centS feature (30).The genes screened by Lasso were scored using the “important” function, and genes with scores above 2 points were selected for further analysis. Multivariate Cox regression analysis was used for final screening and construction of the centrosome signature. The risk score of the signature was defined as the “centrosome score” (centS), which was calculated based on a multivariate Cox regression model: centS = ho(t) * exp (β_1_X_1_+ β_2_X_2_+,…. + β_n_X_n_). In the equation, β refers to the regression coefficient, and ho(t) is the baseline risk function. The patients in the LGG cohort were divided into high- and low-centS groups according to the median centS value. The “ggalluvial” R package was used to draw a Sankey diagram to visualize the correspondence among centS, different subtypes, and prognosis. Univariate or multivariate Cox regression analyses were conducted to confirm the independence of centS in predicting disease prognosis. K-M survival analysis was used to compare the difference in OS between the low- and high-centS groups. Receiver Operating Characteristic (ROC) curves were further generated to examine the efficiency and accuracy of centS in predicting survival outcomes at 1-, 3-, and 5-years.

### Genomic mutation exploration based on centS grouping

Simple nucleotide variation data were downloaded from the GDC database (https://portal.gdc.cancer.gov/). The TMB value was calculated based on these data (TMB (Mut/Mb) = total number of mutations (including synonymous and nonsynonymous point mutations, substitutions, insertions, and deletions)/coding region size of the target region). Difference, correlation, and survival analyses were used to analyze the relationship between TMB, centS, and prognosis. We divided patients of LGG into high TMB (H-TMB) group and low TMB (L-TMB) group based on optimal cutoff, which was determined using the “surv cutpoint” and “surv categorize” functions of the “survminer” R package and was taken into consideration as the boundary. The differences in the genomic mutational landscape in the high- and low- centS groups was explored using the “maftools” R package. In addition, we explored the potential relationship between IDH1 mutations, 1p19q codeletion, MGMTp methylation level, and centS using CGGA cohort data.

### Clinical subgroup analysis based on centS

We selected “age,” “gender,” “grade,” and “histological type” as clinical subgroup characteristics, and analyzed the proportion of each clinical characteristic in the high- and low-centS groups and the differences in centS between different clinical characteristics. Survival curves showed the effect of centS on the prognosis of patients with LGGs of different grades.

### Prediction of chemoradiotherapy efficacy using centS

We extracted samples of WHO II-III grade in CGGA and analyzed the survival differences of high- or low-centS in the TMZ therapy, radiotherapy, and TMZ combined with radiotherapy groups. We then used K-M survival analysis to explore patient prognosis under different treatment combinations (non-TMZ+non-Radio, non-TMZ+Radio, TMZ+non-Radio, and TMZ+Radio) based on centS.

### Evaluation of tumor immune microenvironment infiltration based on centS

The proportions of stromal and immune components were represented using the StromalScore and ImmuneScore, respectively. The estimate score is the product of the StromalScore and ImmuneScore, and it is inversely associated with tumor purity (31). We used the “ESTIMATE” R package to calculate the StromalScore, ImmuneScore, and ESTIMATEScore. We analyzed the correlation between the model genes centS and immune checkpoint genes, and visualized it in a heat map. The relative abundance of each immune cell infiltration was determined using single-sample Gene Set Enrichment Analysis (ssGSEA) using the “GSVA” R package (32). Immune cell infiltration levels were evaluated using ssGSEA, and a correlation heat map was created to show the connection between centS and immune cell infiltration levels. Furthermore, we used the IMvigor210 cohort to investigate the effect of centS on immunotherapy outcomes.

### Analysis of TMZ sensitivity based on centS and identification of the key gene CEP135

Using “cgp2016” as the reference dataset, we constructed a ridge regression model to predict the IC50 value of TMZ in the high- and low-centS groups using the pRRopheticPredict function of the “pRRophetic” R package. The relationship between the 13 CRGs and 12 signature genes and TMZ sensitivity was visualized using a matrix of correlation. Univariate Cox regression analysis and area under the ROC curve (AUC) for single-gene survival analysis were used to identify the key genes. We selected the gene with the greatest risk ratio as the key gene using AUC>=0.8 as the inclusion criterium.

### Screening and validation of the potential CRGs

Potential CRGs were screened using gene co-expression network analysis of the key gene CEP135 and signature genes, with a correlation coefficient of r>0.5 as the standard. We then selected three genes that were most relevant to the key genes as potential CRGs. Finally, DGE analysis of the potential CRGs with the core gene CEP135 was performed using qRT-PCR.

### Cell lines and cell cultures

Human astrocytes (NHA) and astroglioma cells (SW1088) were obtained from the American Type Culture Collection (Manassas, VA, USA). The oligodendroglioma cells (HS683) were purchased from the Cell Bank of Type Culture Collection of the Chinese Academy of Science (Shanghai, China). HS683 cells were cultured in Dulbecco’s modified Eagle’s medium (DMEM; HyClone, Logan, USA), supplemented with 10% fetal bovine serum (FBS; Gibco, NY, USA) and 1% penicillin-streptomycin (HyClone, Logan, USA). SW1088 cells were cultured in Leibovitz’s (L)- 15 medium supplemented with 10% FBS (Gibco, NY, USA) and 1% penicillin-streptomycin (HyClone, Logan, USA). NHA cells were cultured in DMEM (HyClone, Logan, USA) supplemented with 15% FBS (Gibco, NY, USA) and 1% penicillin-streptomycin (HyClone, Logan, USA). All the cells were cultured at 37°C in an incubator with 5% CO_2_.

### Quantitative reverse transcription polymerase chain reaction

Total RNA was extracted using the Trizol reagent (Invitrogen). The PrimeScript RT Reagent Kit (TaKaRa, Shiga, Japan) was used to reverse transcribe 1 µg of total RNA into cDNA, and the SYBR Green PCR Master Mix was used for qRT-PCR. Relative gene expression was calculated using the 2–ΔΔCT method, with GAPDH as an internal control. Visualization of qRT-PCR results and two-sample unpaired t-tests were performed using GraphPad Prism version 9.0.1. Primer sequences are listed in **Supplementary Table 1**.

### Statistical analysis

Perl language was used for data processing and R 4.2.1 was used to conduct all analyses. Correlation analysis was performed using Spearman’s and Pearson’s correlation coefficients. The Wilcoxon test was applied using the “limma” R package for DGEs analysis to compare the two groups in the bioinformatic analysis part. The Student’s t-test was performed for the DGEs analysis in the experimental part to compare the two groups. The Kruskal-Wallis test was used for comparisons involving more than two groups. The survival of the different groups was compared using K-M survival analysis and the log-rank test. A two-tailed p value of <0.05 was considered statistically significant.

## Results

### Variation, expression, and survival analysis of CRGs in LGG

**Figure 1:**
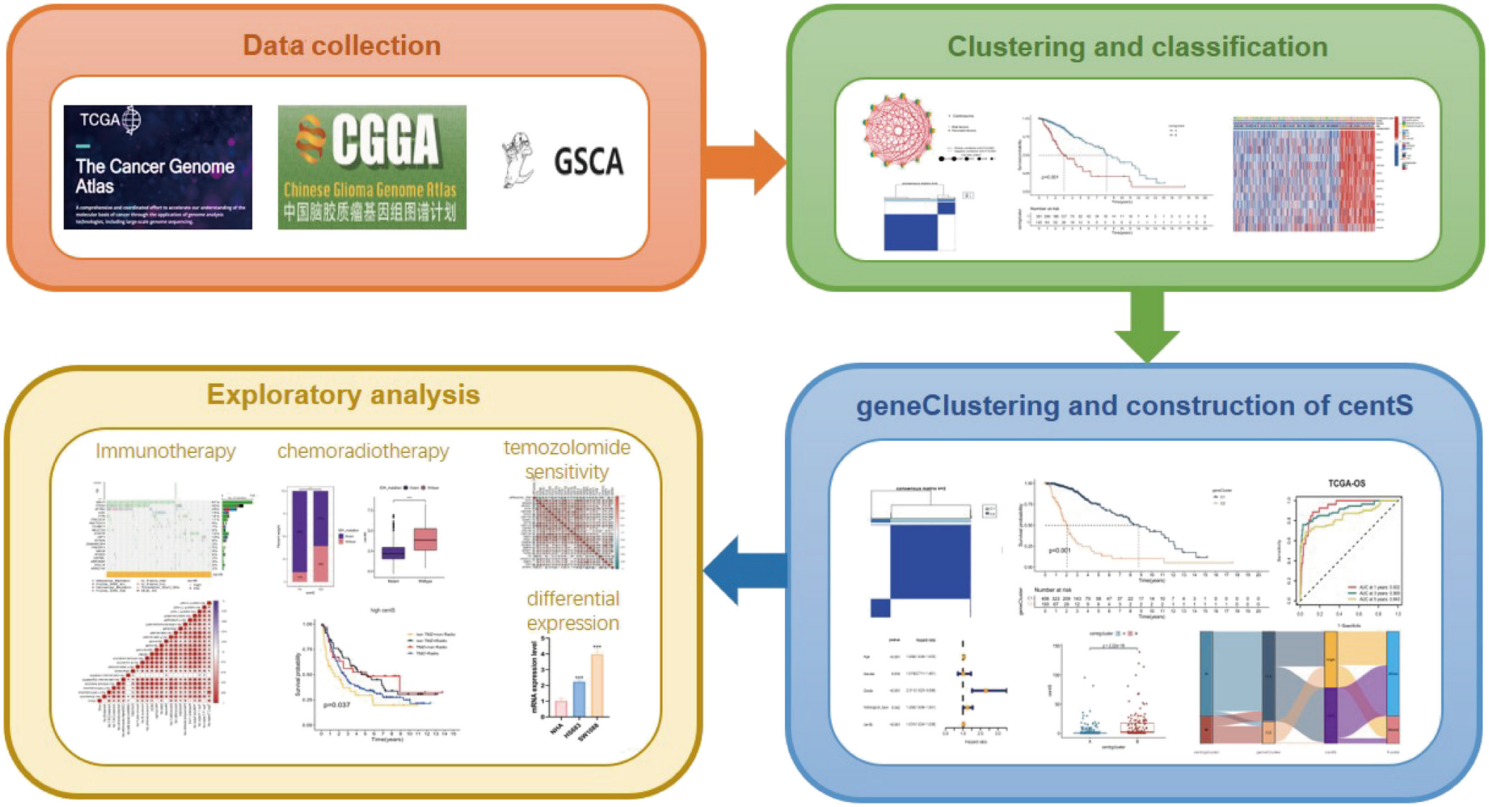
The workflow of this study.

**Figure 2:**
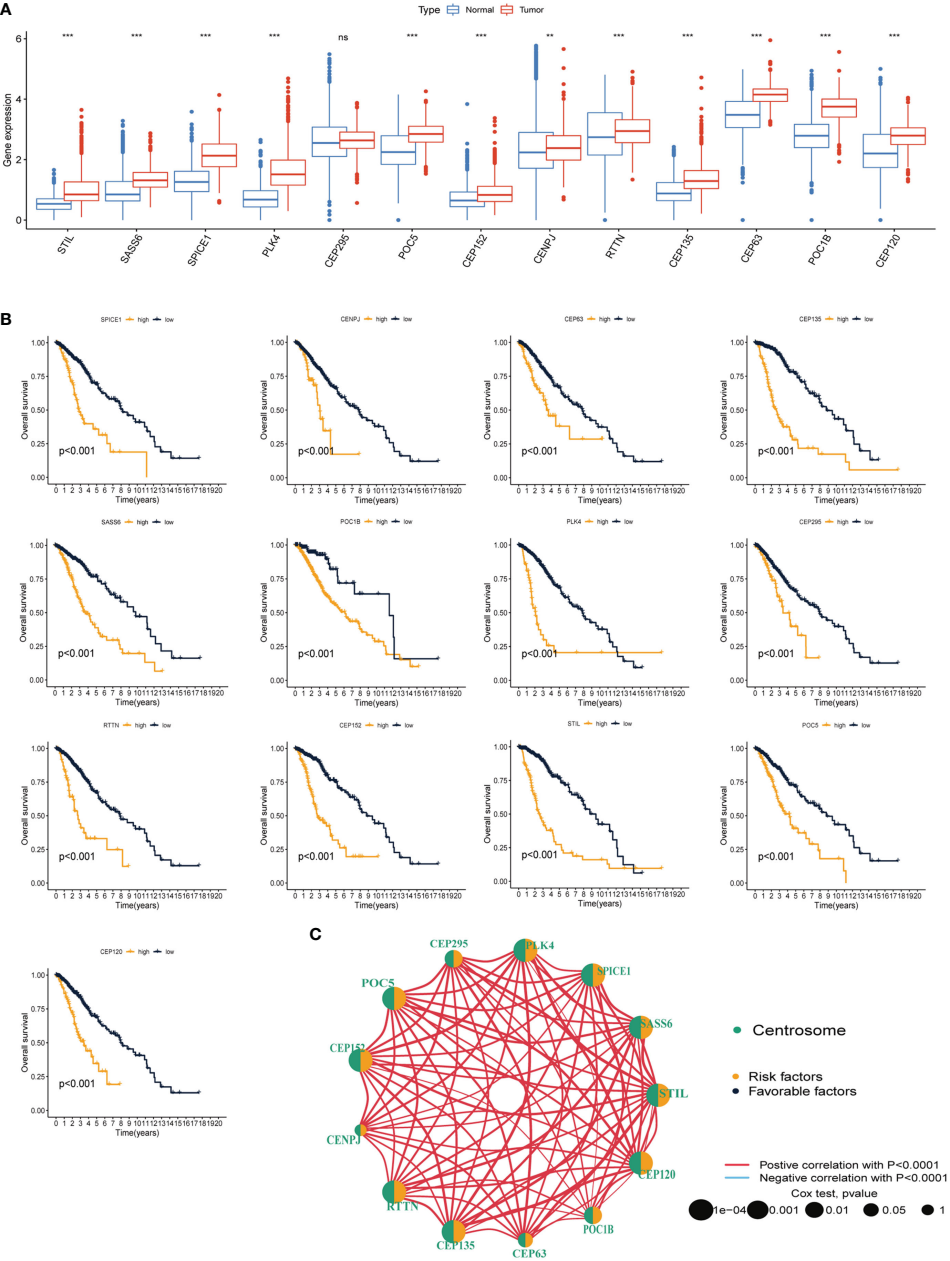
Differential expression and prognostic analysis of thirteen CRGs in TCGA-LGG cohort. **(A)** Differential expression of thirteen CRGs in LGG and normal tissue. **(B)** K-M survival analysis of thirteen CRGs in LGG. **(C)** Correlation network analysis of thirteen CRGs. The line represents the correlation between genes, the sphere represents the univariate Cox test of each gene. CRGs, centrosome related genes; **p < 0.01, ***p < 0.001, ns, not statistically significant.

The workflow of this study is illustrated in **Figure 1**. We conducted a variation analysis of the 13 CRGs using the GSCA database. **Supplementary Figure 1A** shows the chromosomal distribution of CRGs. The SNV frequencies of the ten CRGs are displayed in the form of a heat map (no data are available for SSAS6, CEP295, and POC5), and RTTN had the greatest SNV frequency (**Supplementary Figure 1B**). All 13 CRGs had CNV gain and loss frequency in different degrees, with CEP135 having a very large percentage of insertion mutations and CENPJ having the highest frequency of deletion mutations (**Supplementary Figure 1C**). The CNV of CRGs were positively correlated with their mRNA expression, especially that of SSAS6 (**Supplementary Figure 1D**). We then constructed the mutational landscape of 13 CRGs in the TCGA-LGG cohort, and RTTN had the highest mutation frequency, with missense mutations being the most common mutation type (**Supplementary Figure 1E**). Next, we explored the differential expression of 13 CRGs in LGG and normal samples combined with TCGA and GTEx databases and discovered that CRGs expression was significantly higher in glioma tissues than that in normal tissues, except for CEP295 (**Figure 2A**). Furthermore, K-M survival analysis revealed that 13 CRGs had substantial impact on the prognosis of patients with LGG (p<0.001, **Figure 2B**). Finally, we constructed a network of 13 CRGs showing the results of the correlation and Cox regression analyses; 13 CRGs were significantly positively correlated with each other (p<0.0001) and were risk factors for LGG (hazard ratio [HR]>1, **Figure 2C**).

### Identification and evaluation of subtypes based on the 13 CRGs

**Figure 3:**
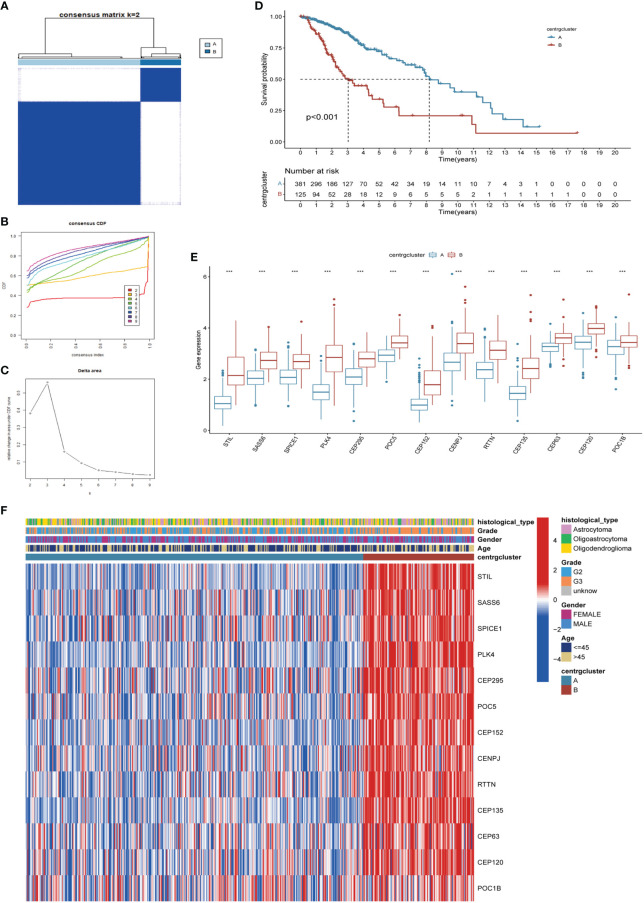
Identification of CRGs subtypes and exploration of the clinical and biological features of subtypes. **(A)** Unsupervised consensus clustering divides LGG samples into two clusters (k=2). Detailed of consensus clustering to identification of CRGs subtypes: cumulative distribution curve **(B)** and area under the cumulative distribution curve **(C)**. **(D)** OS curves of patients with two subtypes of LGG. **(E)** Differential expression of CRGs in different subtypes. **(F)** Clinicopathological characteristics and CRGs expression of two distinct subtypes. CRGs, centrosome related genes; ***p < 0.001.

Unsupervised clustering and classification were performed based on the 13 CRGs. We produced the best classification results when classifying patients with LGG into clusters A and B (**Figures 3A–C**). There was a distinct difference in OS between the two CRGs subtypes, with cluster A having a better prognosis than cluster B (p<0.001, **Figure 3D**). The expression levels of the 13 CRGs were higher in cluster B than those in cluster A (p<0.001, **Figure 3E**). We then created a heat map to show the differences in clinicopathological features and expression distribution of the 13 CRGs in the two CRGs subtypes (**Figure 3F**). We utilized GSVA to compare the variant pathways of the two subtypes from the three sets of the KEGG pathway (**Supplementary Figure 2A**), HALLMARK pathway (**Supplementary Figure 2B**), and Reactome pathway (**Supplementary Figure 2C**) and observed significant differences primarily in multiple cell cycle and cell division pathways. Principal Component Analysis showed that patients in different clusters demonstrated identifiable differences in CRGs expression features (**Supplementary Figure 3A**). We used the ESTIMATE algorithm to assess the immune infiltration state of the two clusters and observed that cluster B had higher StromalScore, ImmuneScore, and ESTIMATEScore than cluster A (**Supplementary Figure 3B**). We used ssGSEA to assess the abundance of 23 infiltrating immune cells and found that the infiltration levels of most immune cells in cluster B were significantly higher than those in cluster A, including CD4 T cells, CD8 T cells, dendritic cells, CD56 dim structural killer cells, gamma delta T cells, natural killer T cells, type 1 T helper cells, and type 2 T helper cells (p<0.05, **Supplementary Figure 3C**).

### Identification and enrichment analysis of DEGs and secondary clustering

**Figure 4:**
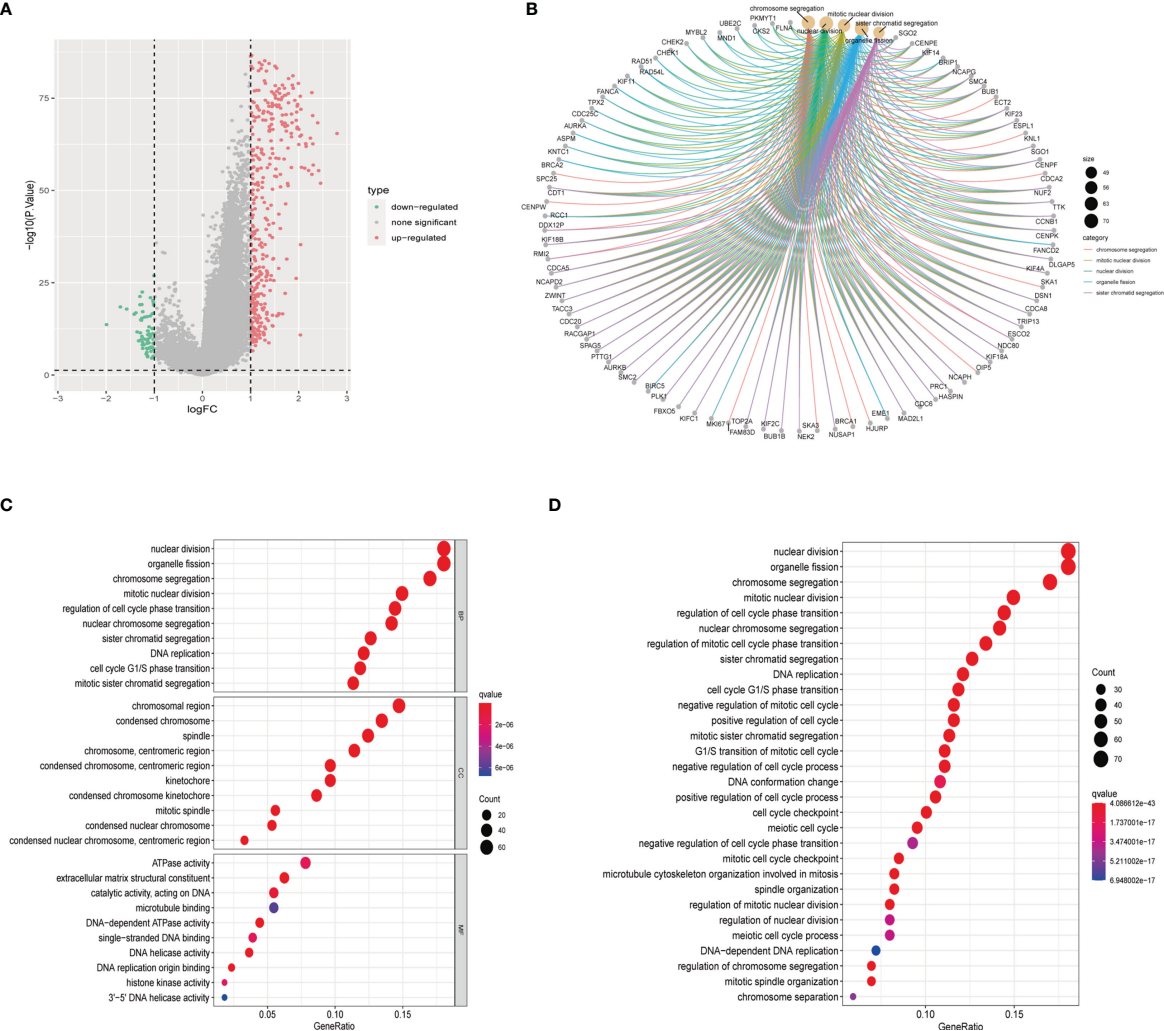
Identification and enrichment analysis of DEGs from two CRGs subtypes. **(A)** Volcano plot of difference analysis between the two CRGs subtypes to identify DEGs. **(B)** The network map displays the correspondence between the KEGG top five pathways and related genes. **(C)** GO enrichment analysis of DEGs. BP, Biological Process; CC, Cellular Component; MF, Molecular Function **(D)** KEGG enrichment analysis of DEGs. CRGs, centrosome related genes; DEGs, differentially expressed genes.

**Figure 5:**
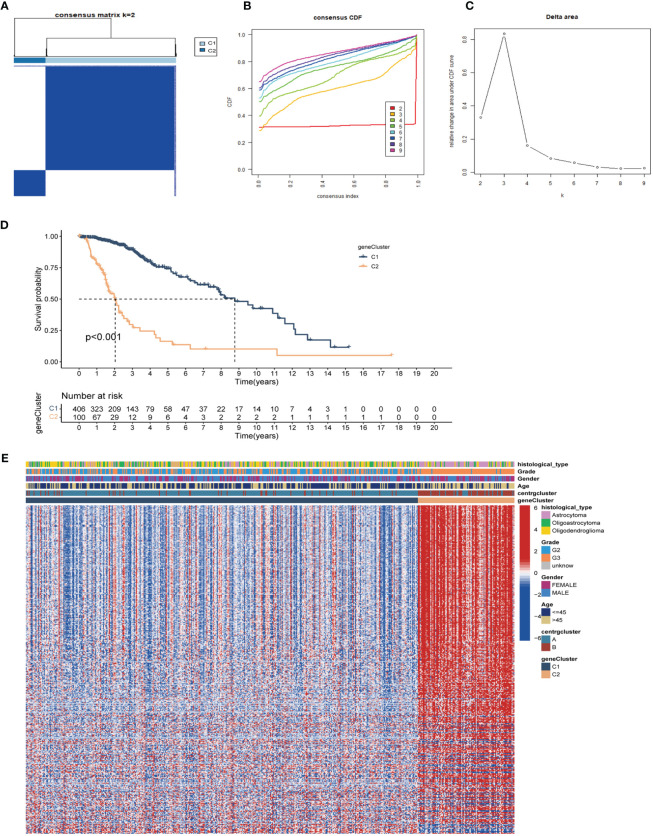
Unsupervised clustering analysis based on prognostic DEGs. **(A)** Consensus clustering divides LGG samples into two gene-subtypes (C1 and C2) base on DEGs (k=2). Detailed of consensus clustering to identification of DEGs subtypes: cumulative distribution curve **(B)** and area under the cumulative distribution curve **(C)**. **(D)** OS curves of patients in C1 and C2 gene-subtypes. **(E)** Clinicopathological characteristics and expression of differential genes of C1 and C2. DEGs, differentially expressed genes.

We conducted DGEs analysis between clusters A and B to further investigate their probable biological functions. We identified 427 DEGs with | logFoldChange|>1 and p<0.05, as shown in the volcano plot (**Figure 4A**). GO (**Figure 4C**) and KEGG (**Figure 4D**) enrichment analyses were performed based on the DEGs, and the top five pathways in the KEGG analysis were nuclear division, organelle fission, chromosome segregation, mitotic nuclear division, and regulation of cell cycle phase transition. Their relationship networks with related genes are shown in **Figure 4B**. Univariate Cox regression analysis was performed on 427 DEGs, of which 407 were found to be significantly related to LGG prognosis. We then performed clustering again and identified two clusters, C1 and C2, based on the expression of the 407 DEGs (**Figures 5A–C**). Survival analysis showed that C1 patients had a better prognosis than C2 patients (**Figure 5D**). The clinicopathological features and expression distribution of DEGs showed significant differences between C1 and C2 on the heat map (**Figure 5E**).

### Construction and evaluation of the centrosome signature

**Figure 6:**
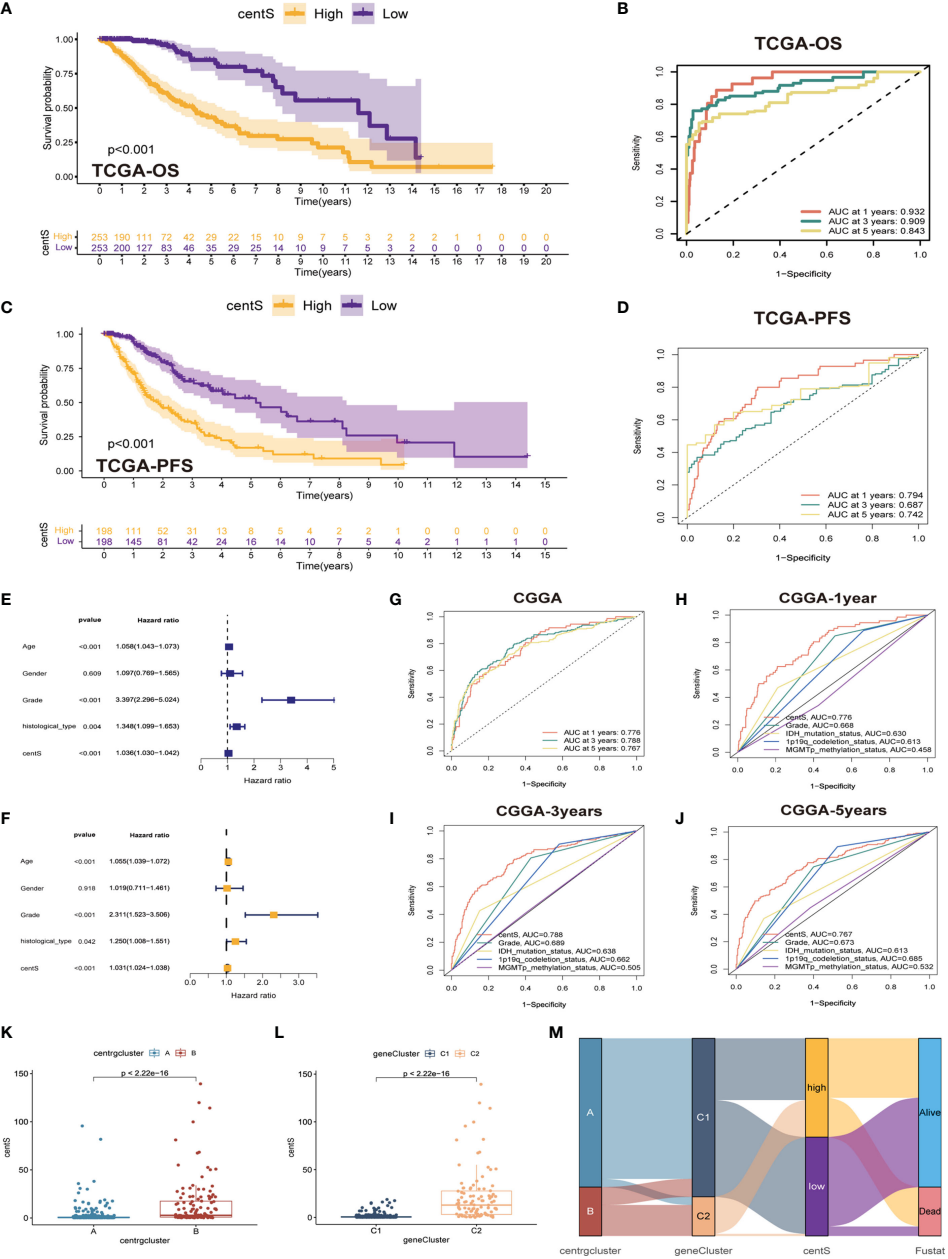
Construction and validation of centS and grouping based on centS. **(A)** OS analysis between the high- and low-centS groups in TCGA cohort. **(B)** ROC curves of centS in predicting OS at 1-, 3-, and 5-years in TCGA cohort. **(C)** PFS analysis between the high- and low-centS groups in TCGA cohort. **(D)** ROC curves of centS in predicting PFS at 1-, 3-, and 5-years in TCGA cohort. **(E, F)** Verification of the clinical independence of centS by univariate Cox regression analysis **(E)** and multivariate Cox regression analyses **(F)**. **(G)** ROC curves of centS in predicting OS at 1-, 3-, and 5-years in CGGA cohort. **(H–J)** ROC curves of centS and other LGG prognostic molecules (grade, IDH mutation status, 1p19q codeletion status, and MGMTp methylation status) in predicting OS at 1-, 3-, and 5-years in CGGA cohort. **(K)** Differences in centS levels between the two CRGs subtypes. **(L)** Differences in centS levels between the two gene-subtypes. **(M)** Sankey diagram to show the correspondence among subtypes, gene-subtypes, centS and survival outcomes. centS, centrosome score; CRGs, centrosome related genes; DEGs, differentially expressed genes.

To assess the predictive value of the centrosome-related DEGs on clinical prognostic characteristics and treatment outcomes in patients, we performed Lasso Cox regression analysis and random forest analysis to screen characteristic genes from the 407 prognostic DEGs and obtained 26 characteristic DEGs (**Supplementary Figures 4A–D**). Subsequently, we performed multivariate Cox regression analysis on the 26 DEGs to obtain 12 model genes to construct the centrosome signature and calculated the centS for each sample (**Supplementary Table 2**). The expression of the 12 signature genes in LGG tissues was higher than that in normal glial tissues (**Supplementary Figure 5A**). The K-M survival curve showed that most of the signature genes were associated with poor prognosis in LGG patients, while the opposite was demonstrated for F5 and SFRP2 (**Supplementary Figure 5B**). Next, we divided samples into high- and low-centS groups depending on the median value of centS, “0.6246025”. The K-M survival analysis revealed that patients in the high-centS group had a poorer prognosis than those in the low-centS group (**Figure 6A**). The ROC curve showed that centS had excellent predictive ability for the prognosis of LGG patients, the AUC values at 1-, 3-, and 5-years were 0.932, 0.909, and 0.843, respectively (**Figure 6B**). Patients in the high-centS group also had shorter PFS (**Figure 6C**), and the AUC values of PFS at 1-, 3-, and 5-years were 0.794, 0.687, and 0.742, respectively (**Figure 6D**). Univariate and multivariate Cox regression analyses indicated that centS was an independent prognostic factor (**Figures 6E, F**). Furthermore, we verified this conclusion in the CGGA cohort, the ROC curve illustrated that centS had a good predictive performance for the prognosis of patients with LGG, and the AUC values of OS at 1-, 3-, and 5-years were 0.776, 0.788, and 0.767, respectively (**Figure 6G**). The AUC values of centS at 1- (**Figure 6H**), 3- (**Figure 6I**), and 5-years (**Figure 6J**) were larger than grade, IDH mutation status, 1p19q codeletion status, and MGMTp methylation status. A Sankey diagram was drawn to illustrate the link between subtypes, gene subtypes, centS, and prognosis (**Figure 6M**). Patients with LGG in subtype B were more likely to match the gene subtype C2, which has a higher centS and worse prognosis. This conclusion was also supported by the box plots (**Figures 6K, L**).

### Clinical subgroup analysis based on centS

To further investigate the association between centS and normal LGG clinical characteristics, we used stacked histograms to display the percentage of each clinical characteristic in the high- and low-centS groups, and box plots to display the centS difference among various clinical characteristics. Patients older than 45 years had higher centS (**Supplementary Figure 6A**), and there was no significant difference between males and females (**Supplementary Figure 6B**). Patients with histological grade G3 had higher centS than those with histological grade G2 (**Supplementary Figure 6C**). In both G2 and G3 phases, patients in the high-centS group had a poor prognosis (**Supplementary Figures 6E, F**) and patients with astrocytoma type had a higher centS than those with oligoastrocytoma and oligodendroglioma types (**Supplementary Figure 6D**). Moreover, we extracted information on patients with LGG recurrence and analyzed the relationship between centS and LGG recurrence. We discovered that there was a higher percentage of patients with recurrence in the high-centS group (**Supplementary Figure 6G**), and at the same time, the patients with recurrence had a higher centS (**Supplementary Figure 6H**).

### Mutation analysis and tumor immune microenvironment analysis

**Figure 7:**
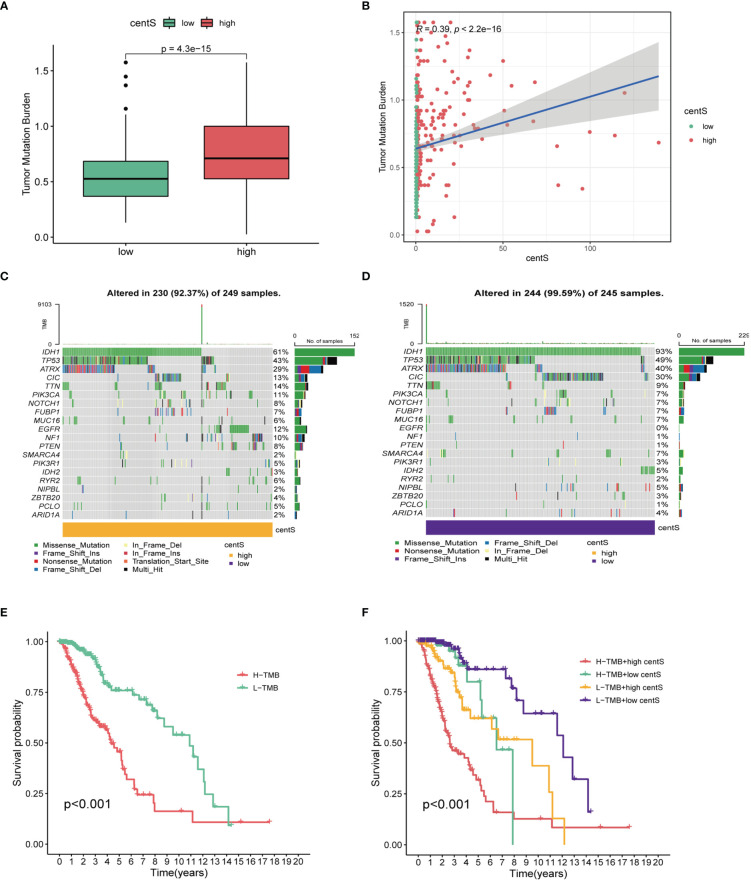
Tumor mutation analysis base on centS in TCGA-LGG cohort. **(A)** Difference analysis of TMB between the high- and low- centS groups. **(B)** Correlation analysis between centS and TMB. **(C, D)** Tumor mutation landscape in the high- and low- centS groups. **(E)** OS analysis of patients with high- and low- TMB. **(F)** OS analysis of patients in different combination groups of centS and TMB. centS, centrosome score.

**Figure 8:**
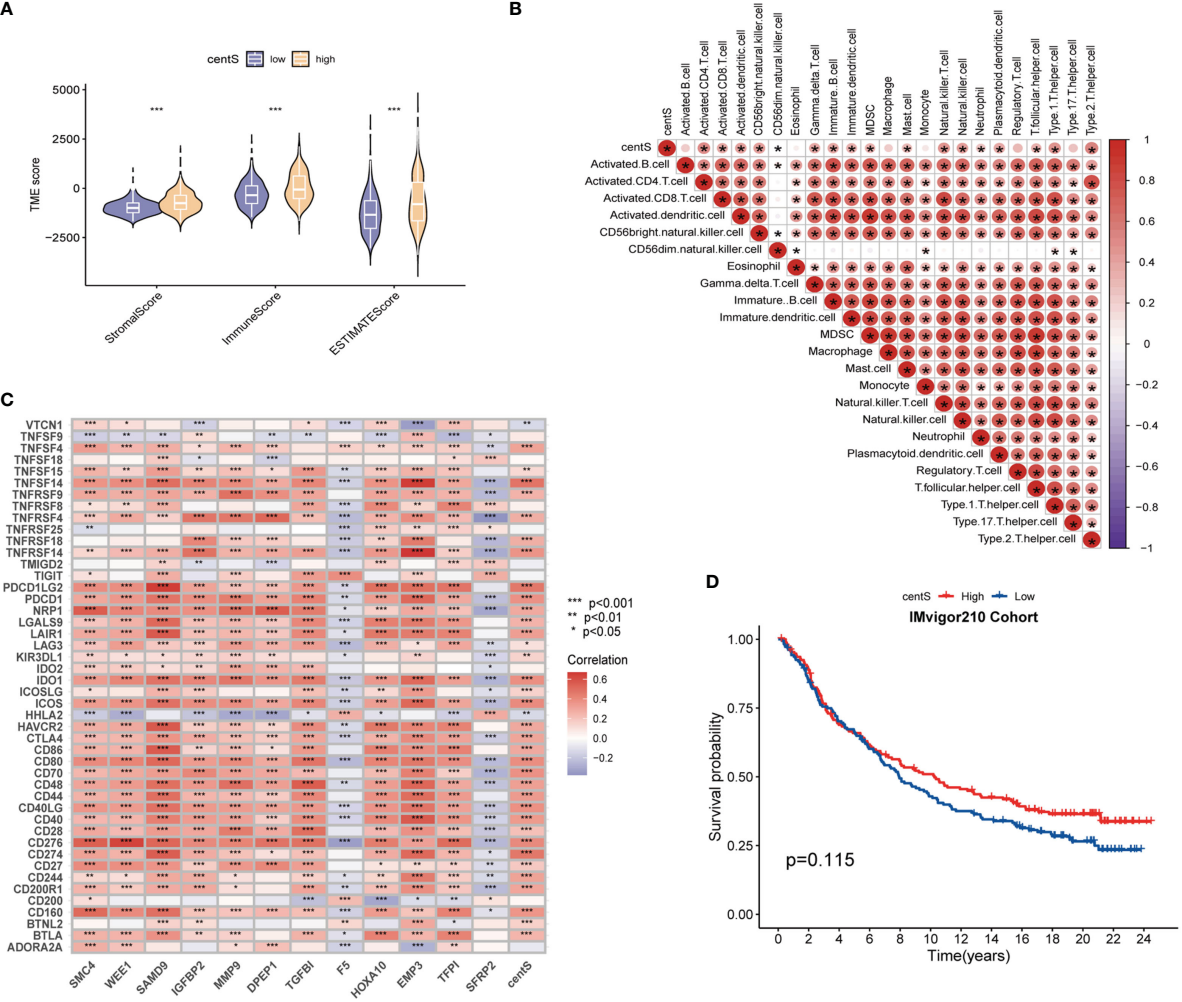
Tumor Immune microenvironment analysis base on centS. **(A)** Violin diagram for difference comparison of StromalScore, ImmuneScore, and ESTIMATEScore in the low- and high-centS groups. **(B)** Correlation heat map of centS and 23 immune cells based on ssGSEA. **(C)** Co-expression heat map of the 12 signature genes and centS with 46 ICGs. **(D)** OS analysis based on centS grouping in IMvigor210 Cohort. centS, centrosome score; ICGs, immune checkpoint genes. *p < 0.05; **p < 0.01; and ***p < 0.001.

The TMB, which is measured as the number of somatic coding mutations per megabase (mutations/Mb) of the tumor genotype (33), has emerged as a useful biomarker for predicting the effectiveness of immunotherapy in a variety of cancer types (34). We found that the high-centS group had higher TMB (**Figure 7A**), and there was a positive correlation between centS and TMB (**Figure 7B**), while IDH1, TP53, ATRX, and IDH2 had lower mutation frequencies in the high-centS group than in the low-centS group (**Figures 7C, D**). The results of the OS analysis showed that individuals with high TMB had poorer prognosis than those with low TMB (**Figure 7E**). The cohort with high TMB combined with high centS had the worst prognosis, whereas the group with low TMB combined with low centS had the best prognosis (**Figure 7F**). Next, we explored the relationship between immune infiltration, immunotherapy, and centS in patients with LGG. The violin diagram illustrated that the high-centS group had higher StromalScore, ImmuneScore, and ESTIMATEScore compared to the low-centS group (**Figure 8A**). We generated a correlation heat map to investigate the connection between centS and immune cell infiltration, and noticed a positive relationship between centS and the majority of immune cells (**Figure 8B**). Correlation analysis of centS and positively associated immune cells is shown in **Supplementary Figure 7**. Subsequently, the correlation heat map showed the co-expression relationship between 12 signature genes, centS, and 46 immune checkpoint genes (ICGs) (**Figure 8C**). CentS and the majority of the signature genes were positively related to ICGs, whereas F5 and SFRP2 were negatively related to ICGs. Finally, we probed the predictive ability of centS for immunotherapy efficacy using the IMvigor210 cohort, and found that individuals with higher centS are likely to have better immunotherapeutic effects (**Figure 8D**).

### Exploration of antitumor therapy efficacy based on centS

**Figure 9:**
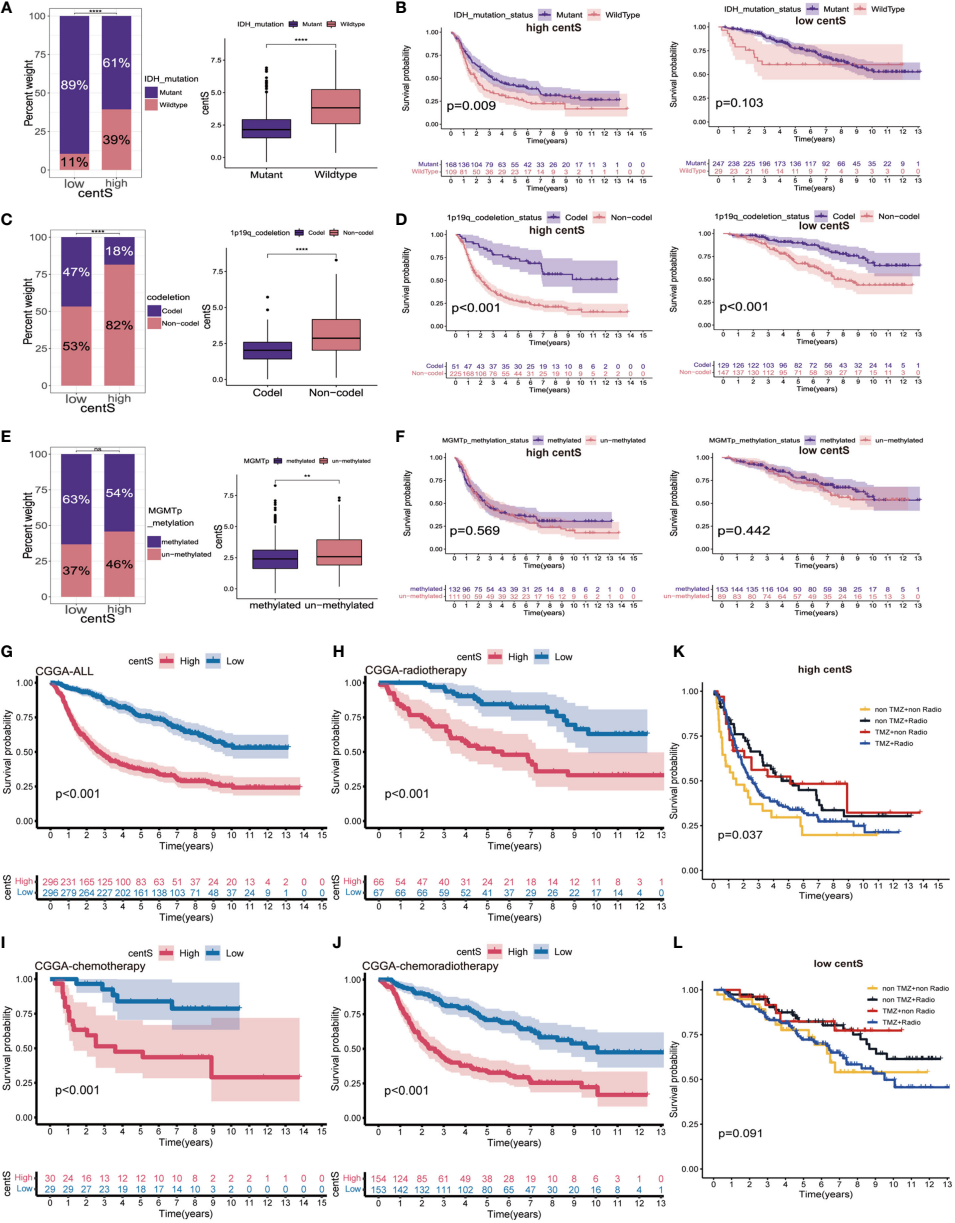
The potential association of centS with genetic status and therapy methods in LGG. Statistical histogram and difference analysis to explore the association of centS with IDH mutation status **(A)**, 1p19q co-deleted status **(C)** and MGMT promoter status **(E)**. OS analysis to show the effects of IDH mutation status **(B)**, 1p19q co-deleted status **(D)** MGMT promoter status **(F)** on prognosis of high- and low- centS groups. OS analysis of high- and low- centS groups in the CGGA cohort **(G)**, CGGA chemotherapy cohort **(H)**, CGGA radiotherapy cohort **(I)**, and CGGA chemo radiotherapy cohort **(J)**. The response of patients with LGG in the high- centS group **(K)** and low- centS group **(L)** to different combination of treatments. centS, centrosome score. **p < 0.01; ****p < 0.0001; "ns", not statistically significant.

Using CGGA-LGG cohort data, we explored the proportion of different IDH mutation status, 1p19q codeletion status, and MGMTp status in the high- and low-centS groups, and the difference in centS among different gene statuses. Our results showed that the high-centS group had a lower proportion of IDH1 mutations (**Figure 9A**) and 1p19q codeletion (**Figure 9C**). There was no significant difference in MGMTp methylation levels between the high- and low-centS groups (**Figure 9E**). Patients with the IDH mutation subtype, 1p19q codeletion subtype, and MGMTp methylation subtype had a lower centS (**Figures 9A, C, E**). We then investigated the effect of different gene statuses on the prognosis of patients with LGG in the high- and low-centS groups. In the high-centS group, the prognosis of patients with the IDH mutant subtype was significantly better than that of patients with the wild-type IDH, but in the low-centS group, the prognosis of the two subtypes was not statistically different (**Figure 9B**). The prognosis of patients with the 1p19q codeletion subtype was remarkably superior to that of patients with the 1p19q non-codeletion subtype in both the low- and high-centS groups (**Figure 9D**). There was no statistical difference in prognosis between the different MGMTp methylation subtypes in both the low- and high-centS groups (**Figure 9F**). Finally, we explored the predictive value of centS in response to chemotherapy, radiotherapy, and chemotherapy combined with radiotherapy. The low-cents group had better survial across different treament cohorts (**Figures 9G–J**). In both the high- and low-centS groups, patients who received radiotherapy alone or chemotherapy alone may have a better prognosis than those who received chemoradiotherapy or no therapy (**Figures 9K, L**).

### TMZ sensitivity analysis and identification of the key gene CEP135

**Figure 10:**
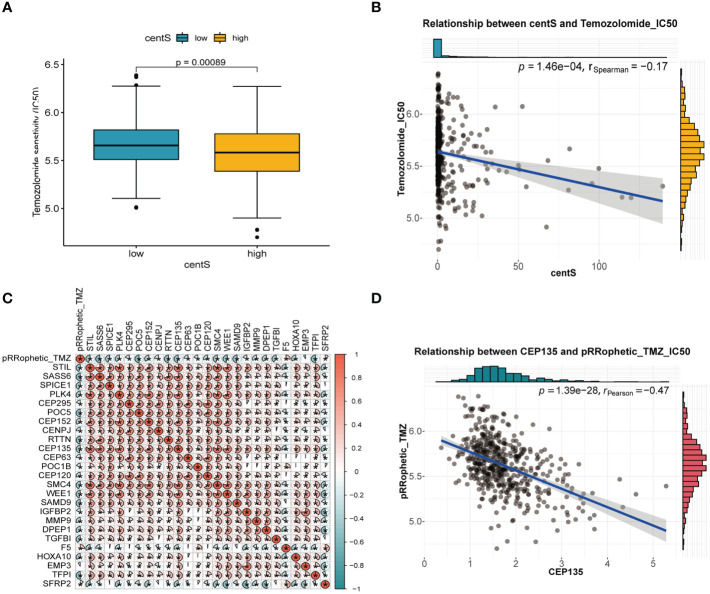
Temozolomide sensitivity analysis of centS and CRGs in LGG. **(A)** difference analysis of IC50 value of Temozolomide in high- and low- centS group performed by “pRRophetic” package. **(B)** Correlation analysis of centS and IC50 value of Temozolomide. **(C)** The correlation matrix to show the relationship of the IC50 value of Temozolomide with 13 CRGs and 12 signature genes. Blue indicates that the gene is negatively correlated with the IC50 of Temozolomide, and red indicates that the gene is positively correlated with the IC50 of Temozolomide. **(D)** Correlation analysis of key gene CEP135 and IC50 value of Temozolomide. *p < 0.05.

We observed lower IC50 value for TMZ in the high-centS group compared with those in the low-centS group, indicating that patients with high centS were more sensitive to TMZ (**Figure 10A**). The correlation scatter plot showed a negative correlation between centS and the IC50 value of TMZ (**Figure 10B**). The correlation matrix visualized the relationship between 13 CRGs, 12 signature genes, and the IC50 value of TMZ, the majority of genes had a negative correlation with the IC50 of TMZ (**Figure 10C**). Then, univariate Cox regression analysis and AUC for single-gene survival analysis were performed to identify the key gene CEP135 (AUC>=0.8, HR=2.64) (**Supplementary Table 3**), the expression of which was negatively related with the TMZ IC50 (**Figure 10D**).

### Identification and expression validation of the potential CRGs team

**Figure 11:**
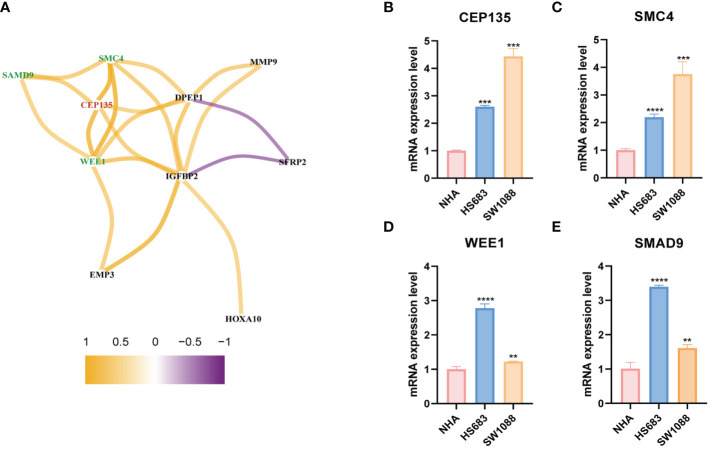
The differential expression verification of potential CRGs team by qRT-PCR. **(A)** The genes co-expressed network of CEP135 and 12 signature genes taking correlation coefficient r>0.5 as the threshold value. red represents the key gene, green represents the three strongest correlation genes with the key gene. **(B–E)** Differential expression verification of CEP135, SMC4, WEE1 and SMAD4 in SW1088, HS683 and NHA cells using qRT-PCR. CRGs, centrosome related genes. **p < 0.01; ***p < 0.001; and ****p < 0.0001. qRT-PCR data are means ± SD, with n = 3.

Based on the gene co-expression network analysis, we identified three genes with the strongest positive correlation with CEP135: structural maintenance of chromosomes 4 (SMC4), WEE1, and human sterile alpha motif protein 9 (SAMD9) (**Figure 11A**). These three genes and CEP135 constitute a potential CRGs team. Subsequently, we detected the expression differences of CEP135 (**Figure 11B**), SMC4 (**Figure 11C**), WEE1 (**Figure 11D**), and SAMD9 (**Figure 11E**) among SW1088, HS683 and NHA using qRT-PCR. The expression of these genes was significantly upregulated in SW1088 and HS683 cells compared to that in NHA cells (p<0.05).

## Discussion

Although it is commonly recognized that LGG has a better prognosis than GBM, LGG still recurs or advances after treatment (35). The centrosome is the main microtubule organization center in animal cells. Centrosomal proteins can modulate the integrity of centrosomes to preserve the stability of microtubules and influence the proliferation and differentiation of neural stem cells (36). In this study, we attempted to classify LGG subtypes based on centrosome-related proteins. Thirteen crucial CRGs were identified and shown to be overexpressed in LGG, indicating that centrosome protein instability may be responsible for the occurrence and progression of LGG.

First, we identified two clusters with different biological and clinical characteristics in the TCGA-LGG cohort, based on 13 CRGs. Compared with cluster A, cluster B had worse prognosis and higher immune cell infiltration levels, including immune-promoting cells, such as CD8+T cells, CD4+T cells, and immune-suppressive cells, such as MDSC. GSVA showed that cluster B was more closely associated with cell cycle-related pathways than cluster A. Next, we analyzed the differences between clusters A and B and identified 427 DEGs. Enrichment analysis showed that the DEGs were associated with various cell cycle and cell division pathways, including chromosome segregation, mitotic nuclear division, nuclear division, sister chromatid segregation, and organelle fission. This demonstrated that we would be able to construct a prognostic model relying on CRGs to assess the therapy and prognosis of LGG, and that targeting centrosome-related proteins may be a viable therapeutic strategy.

Next, we selected 407 DEGs using univariate Cox regression analysis to further construct two new gene clusters. Gene cluster C2 had a poorer prognosis than gene cluster C1. In addition, we selected 12 potential CRGs to establish the centrosome-related risk signature centS to predict the prognosis of LGG and help in clinical decision making. Surprisingly, patients in the high-centS group had a poorer prognosis, and centS showed a high efficiency of prediction.

The TMB has emerged as a valuable biomarker for predicting the efficacy of immune checkpoint blockade in various cancers (34). The TMB is negatively correlated with OS (37), however, patients with high TMB frequently benefit from immunotherapy (38). Immunotherapy is not currently the mainstream treatment for glioma; clinical trials have not produced consistently favorable results in patients with glioma (39), but patients with LGG have shown excellent immune responses to vaccines in previous clinical trial (40), suggesting that immunotherapy may have great potential in LGG. In this study, the high-centS group had a higher TMB, worse prognosis, superior immune cell infiltration level, and better immunotherapy response, compared to the low-centS group. Moreover, the prognosis of the L-TMB combined with the low-centS group was far better than that of the H-TMB combined with the high-centS group. These results indicate the potential predictive value of our signature in the prognosis of LGG and immunotherapy response. Previous studies have shown that IDH1 mutation causes changes in the immune environment; patients with IDH1 mutation have lower expression of PD-L1 (41) and a poor response to tumor immunotherapy (42). Interestingly, the mutation rate of IDH was relatively lower in the high-centS group than in the low-centS group, which also means that the high-centS group has better immunotherapy sensitivity. Our analysis of the IMvigor210 cohort also confirmed this finding. Therefore, immunotherapy may be a promising treatment for LGG in the future, and centS may be an effective marker for predicting response to LGG immunotherapy.

The 2016 WHO included molecular characteristics, such as 1p19q codeletion, IDH, ATRX, and TP53 mutations, in the diagnostic strategy of LGG to offer a more accurate diagnosis (43). The IDH mutation and 1p19q codeletion were more common in the low-centS group than in the high-centS group, and TMB analysis also showed a higher IDH mutation in the low-centS group. Studies have shown that the presence of IDH mutation and 1p/19q codeletion contributes to patient prognosis and prediction of optimal drug therapy (12, 44), which may be one reason why patients with low centS have a better prognosis. In addition, we found that centS could be a better prognostic marker for LGG than traditional IDH mutations, 1p19q codeletion, and MGMT promoter methylation. Of course, centS, in combination with the three traditional genetic markers, may differentiate patients with LGG with different outcomes more accurately. The results of the subgroup survival analysis provide us with a clinical strategy for combining centS and three commonly used glioma molecular indicators for comprehensive prediction. The patients in the high-centS group can continue to detect IDH mutation and 1p19q codeletion to differentiate prognostic risks, while for patients in the low-centS group, only the detection of 1p19q codeletion may lead to new predictive benefits. However, the detection of MGMT promoter methylation is not recommended for further prognostic prediction in either the high- or low-centS group. For high-risk patients with LGG, there is reasonable evidence for adjunctive chemotherapy and radiation therapy after maximal surgical resection (45). We explored the predictive potency of centS for patient survival in different treatment cohorts. Our analysis revealed that regardless of the cohort (chemoradiotherapy, radiotherapy, or radiotherapy combined with chemotherapy cohorts) the low-centS group had a longer survival time. It is worth noting that TMZ chemotherapy or radiotherapy alone can significantly prolong the survival of patients with LGG. Radiotherapy combined with chemotherapy may benefit patients in the high-centS group but has little significance for patients in the low-centS group. As mentioned above, our centrosome-related risk signature may provide a reference for more precise clinical treatment decisions in patients with LGG.

Given that TMZ chemotherapy is still the most effective chemotherapy regimen for high-risk LGG, we explored the relationship between centS and sensitivity to TMZ and found that the high-centS group had a lower IC50 value of TMZ and a higher sensitivity to TMZ than the low-centS group. In addition, almost all CRGs and modeling genes showed a positive correlation with TMZ sensitivity. We speculated that these genes may synergistically enhance sensitivity to TMZ in LGG. We then identified the key gene CEP135 and three of the most potent CRGs with CEP135 from 12 signature genes: SMC4, WEE1, and SAMD9. As a necessary conserved central protein for centrosome replication, an imbalance of CEP135 results in centriole overduplication and contributes to chromosome segregation errors to promote breast cancer (46). Smc4, a core subunit of condensing, has been reported to be associated with a variety of tumors, such as hepatocellular carcinoma (47), prostate cancer (48), and lung adenocarcinoma (49), and activated TGFβ/Smad signaling has been reported to promote the aggressive phenotype of glioma (50). Targeting poly (ADP-ribose) polymerase and cell cycle checkpoints, ATM-CHK2-TP53 and ATR-CHK1-WEE1, can benefit tumor therapy through a synthetic lethality mechanism (51). Moreover, WEE1 inhibition can boost anti-tumor immunity by activating ERV and the dsRNA pathway and strengthen sensitivity to immune checkpoint blockade (52). SAMD9 has been identified as a potential antigen for developing mRNA vaccines against diffuse glioma (53). Furthermore, we verified that these genes were highly expressed in LGG cells compared to normal glial cells using qRT-PCR. In summary, we believe that these four genes may form a gene team that promotes the occurrence and progression of LGG and regulates the sensitivity of LGG to TMZ. Thus, they may be promising therapeutic targets for LGG treatment.

There are a few limitations to our work. On the one hand, the need for an immunotherapy cohort for low-grade gliomas prevents us from further validating and optimizing the predictive value of the scoring system. On the other hand, our current study primarily clarified the predictive ability of centrosome-related gene score on the prognosis and treatment response of low-grade glioma based on bioinformatic analysis, which still requires clinical validation.

## Conclusion

In conclusion, our findings revealed a novel CRG signature named centS that can accurately predict the prognosis of patients with LGG. Furthermore, CentS can assess the patient’s response to TMZ and immunotherapy, helping physicians to develop individualized treatment plans for patients with different genetic statuses.

## Data availability statement

The original contributions presented in the study are included in the article/**Supplementary Material**. The complete original code can be downloaded from "https://www.jianguoyun.com/p/DVCxeRUQ3rToChj8oukEIA". Further inquiries can be directed to the corresponding author.

## Author contributions

KC designed the study. GZ and PT conducted the data analysis, and wrote the manuscript. GZ, PT, AC, and JF participated in the experiment. JF, AC, and XC participated in manuscript revision. All authors contributed to the article and approved the submitted version.

## Glossary

**Table:**

ATRX	alpha-thalassemia/mental retardation, X-linked
AUC	area under the ROC curve
CGGA	Chinese Glioma Genome Atlas
CENPJ	centromere protein J
CEP	centrosomal protein
CNV	copy number variation
CPAP	centrosomal P4.1-associated protein
CRG	centrosome-related gene
DEG	differentially expressed genes
DGE	differential gene expression
DMEM	Dulbecco’s modified Eagle’s medium
FBS	fetal bovine serum
GBM	glioblastoma multiforme
GO	Gene Ontology
GSCA	Gene Set Cancer Analysis
GSVA	Gene Set Variation Analysis
ICG	immune checkpoint genes
IDH	isocitrate dehydrogenase
KEGG	Kyoto Encyclopedia of Genes and Genomes
LASSO	least absolute shrinkage and selection operator
LGG	low-grade gliomas
MGMTp	O^6^-methylguanine-DNA methyltransferase promoter
OS	overall survival
PFS	progression-free survival
PLK4	polo-like kinase 4
POC1B	POC1 centriolar protein
ROC	Receiver Operating Characteristic
RTTN	rotatin
SAS6	spindle assembly abnormal protein 6 homolog
SNV	single nucleotide variation
SPICE1	spindle and centriole associated protein 1
STIL	SCL/TAL interrupting locus
TCGA	The Cancer Genome Atlas
TMB	tumor mutation burden
TMZ	temozolomide
TPM	transcripts per kilobase million
WHO	World Health Organization.
