# Supplementary material for: Molecular subtypes based on centrosome-related genes can predict prognosis and therapeutic responsiveness in patients with low-grade gliomas
Source: Front Oncol. 2023 Mar 27;13:1157115. doi: 10.3389/fonc.2023.1157115 (PMC10083401; doi:10.3389/fonc.2023.1157115)
Supplement: Supplementary file 1 [file DataSheet_1.docx]

Supplementary Material

**Molecular subtypes based on centrosome-related genes can predict prognosis and therapeutic responsiveness in patients with low-grade glioma**

**Ganghua Zhang,^1^** ^†^ **Panpan Tai,^1^** ^†^ **Jianing Fang,^1^ Aiyan Chen,^1^ Xinyu Chen,^1^ Ke Cao^1*^**

^1^Department of Oncology, Third Xiangya Hospital of Central South University, Changsha, 410013, China

^†^ These two authors contributed equally to this work.

*** Correspondence:**Corresponding author: Ke Cao, Department of Oncology, Third Xiangya Hospital of Central South University, 138 Tongzipo Road, Changsha 410013, China

E-mail: csucaoke@163.com

Tel: +86 0731-88618240

Fax: +86 0731-88618285

# Supplementary Data

Supplementary Material should be uploaded separately on submission. Please include any supplementary data, figures and/or tables.

Supplementary material is not typeset so please ensure that all information is clearly presented, the appropriate caption is included in the file and not in the manuscript, and that the style conforms to the rest of the article.

# Supplementary Figures and Tables

For more information on Supplementary Material and for details on the different file types accepted, please see [here](https://www.frontiersin.org/guidelines/author-guidelines#supplementary-material).

## Supplementary Figures


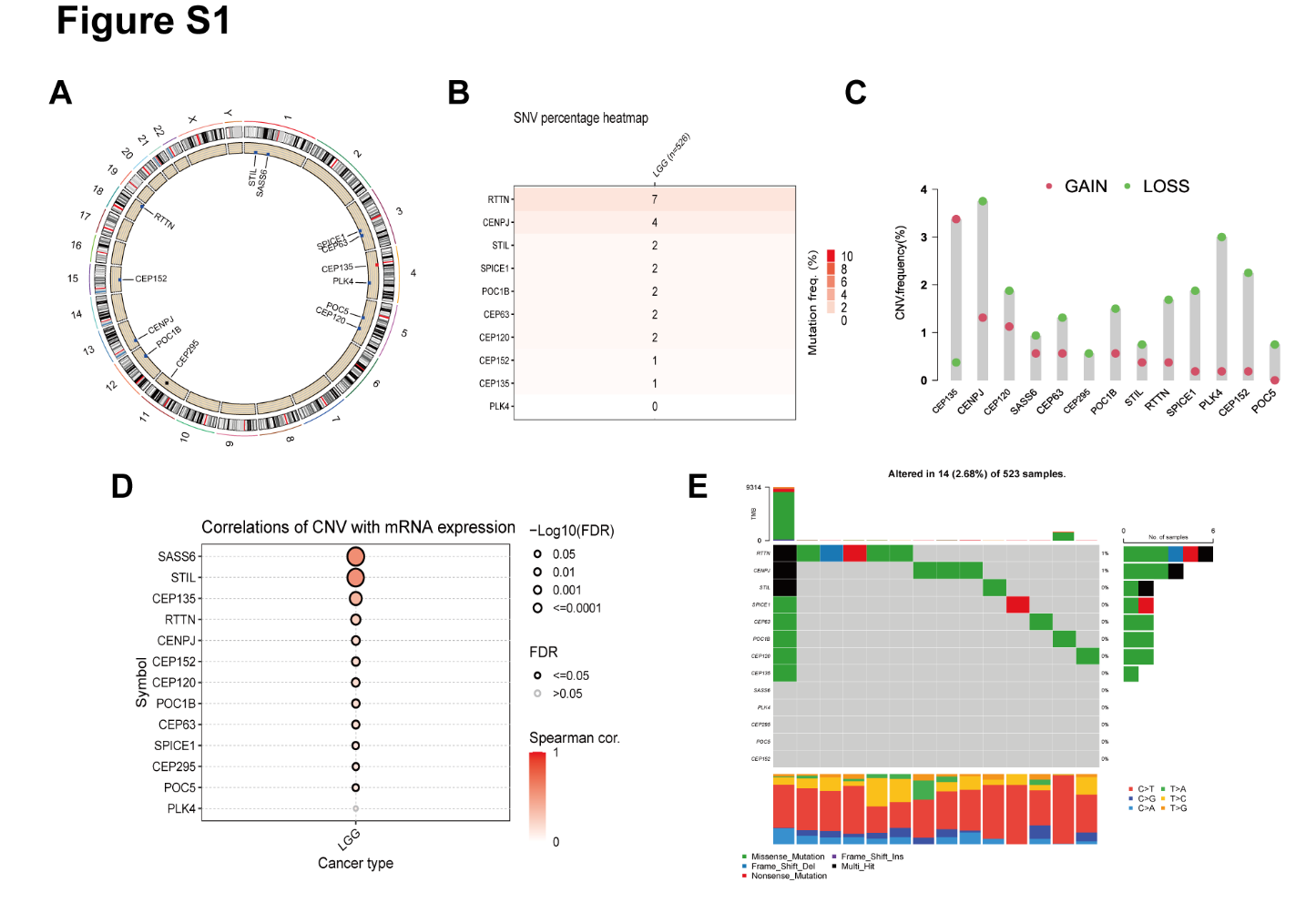


**Supplementary Figure 1.** Genetic alterations of CRGs in LGG in TCGA and GSCA database. (**A**) Localization of thirteen CRGs on chromosomes. (**B**) Frequencies of SNV in CRGs based on GSCA database. (**C**) Frequencies of gain and loss of CNV -in CRGs. (**D**) Correlation between CNV and mRNA expression levels of CRGs in GSCA database. (**E**) Different alteration types and frequencies of CRGs in TCGA-LGG cohort. CRGs, centrosome related genes.


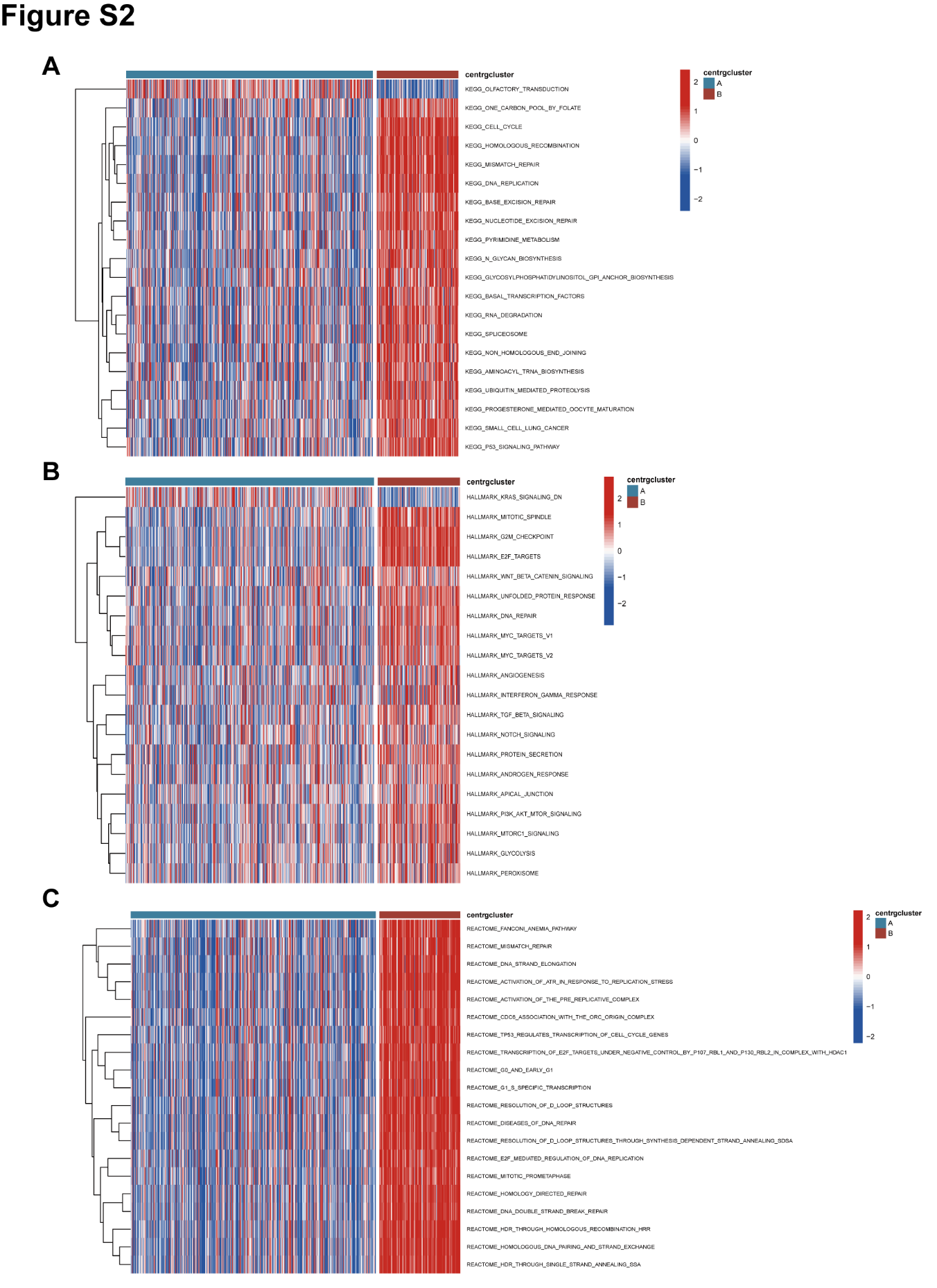


**Supplementary Figure2**. The variant pathways exploration of the two CRGs subtypes by GSVA. KEGG pathway (**A**), HALLMARK pathway (**B**), and Reactome pathway (**C**) analysis of the two subtypes. CRGs, centrosome related genes.


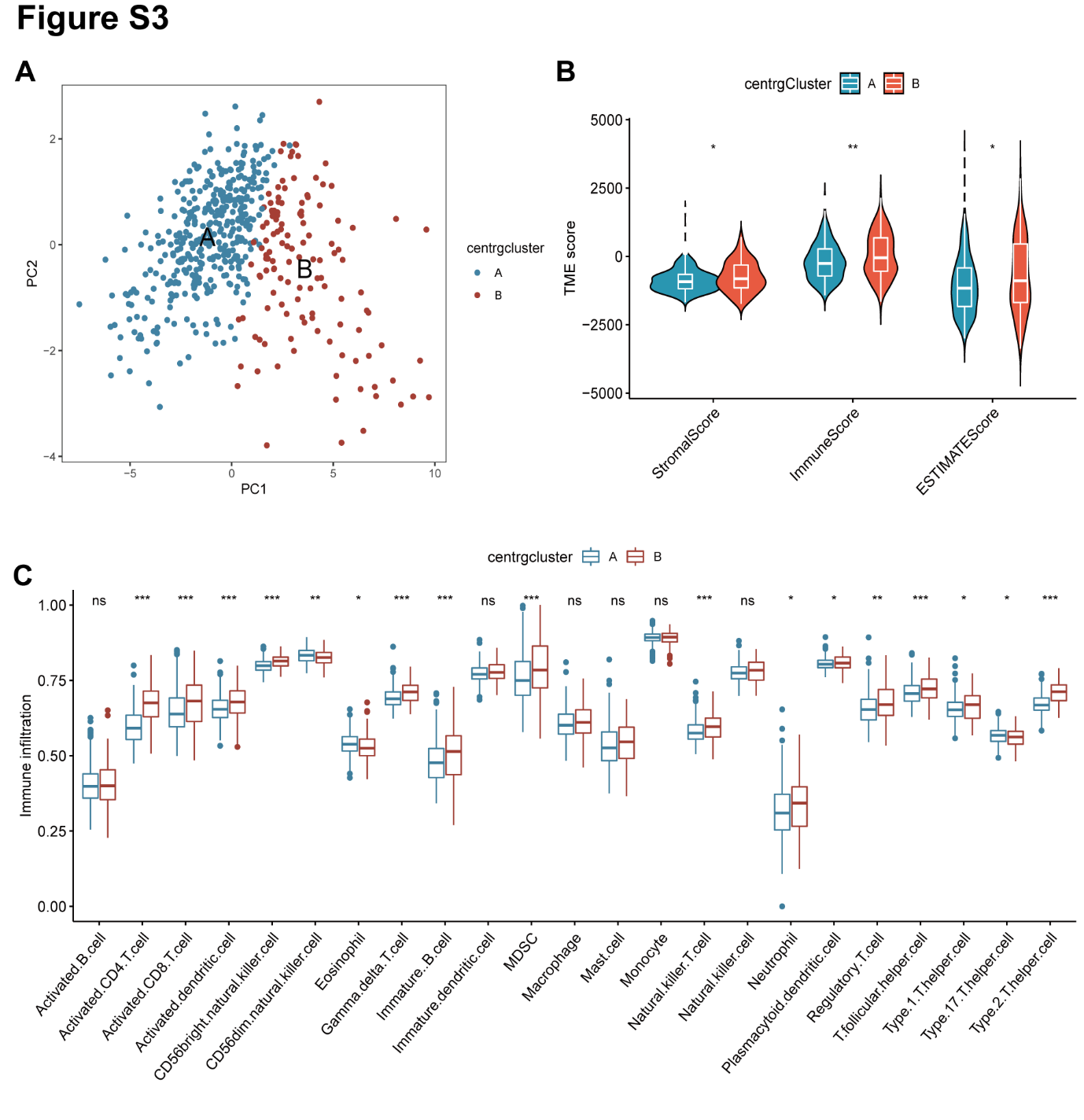


**Supplementary Figure3**. Tumor microenvironment analysis of two CRGs subtypes. (**A**) PCA of the two subtypes based on CRGs expression. (**B**) difference analysis of tumor microenvironment estimate scores between the two CRGs subtypes. (**C**) The abundance of 23 kinds of infiltrating immune cells was evaluated by ssGSEA in the two CRGs subtypes. CRGs, centrosome related genes, *p < 0.05; **p < 0.01; and ***p < 0.001.


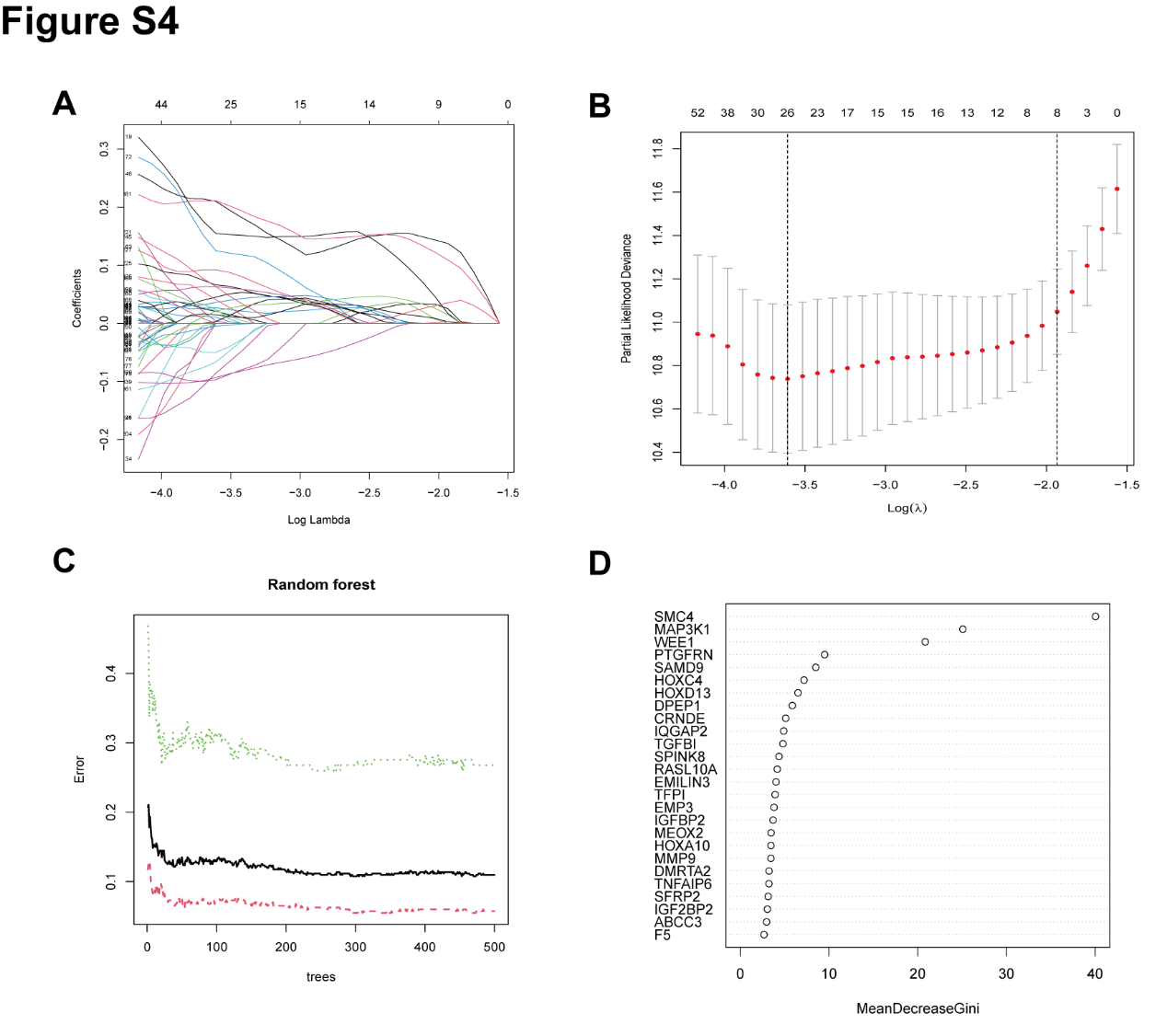


**Supplementary Figure4**. Screening signature genes based on prognostic DEGs. (**A**) Lasso coefficient profiles of the 407 prognostic DEGs. (**B**) Screening of optimal parameter (lambda) and CRGs with non-zero coefficients. When the number of genes with non-zero coefficients was 26, the Lasso model was the most stable. (**C**) The relationship between the number of trees and model error in random forest. The model has the smallest error when the number of trees is 294. (**D**) The top 30 genes were ranked by gene importance score based on Gini coefficient method. DEGs, differentially expressed genes.

**
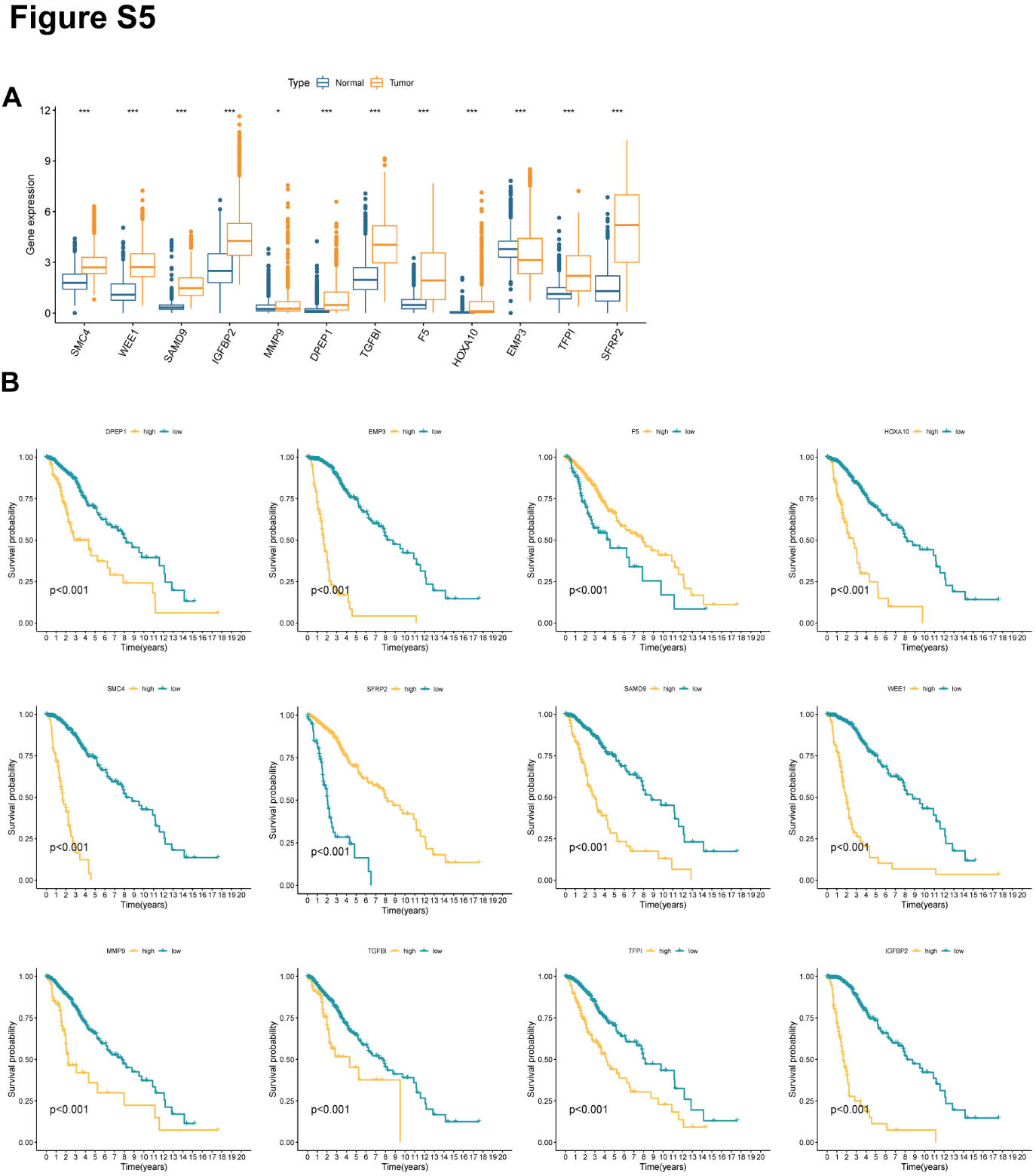
**

**Supplementary Figure5**. Differential expression and prognosis analysis of 12 signature genes in LGG. (**A**) Differential expression of 12 signature genes in LGG tissue and normal tissue based on TCGA and Gtex database. (**B**) K-M survival analysis of 12 signature genes in TCGA-LGG cohort.


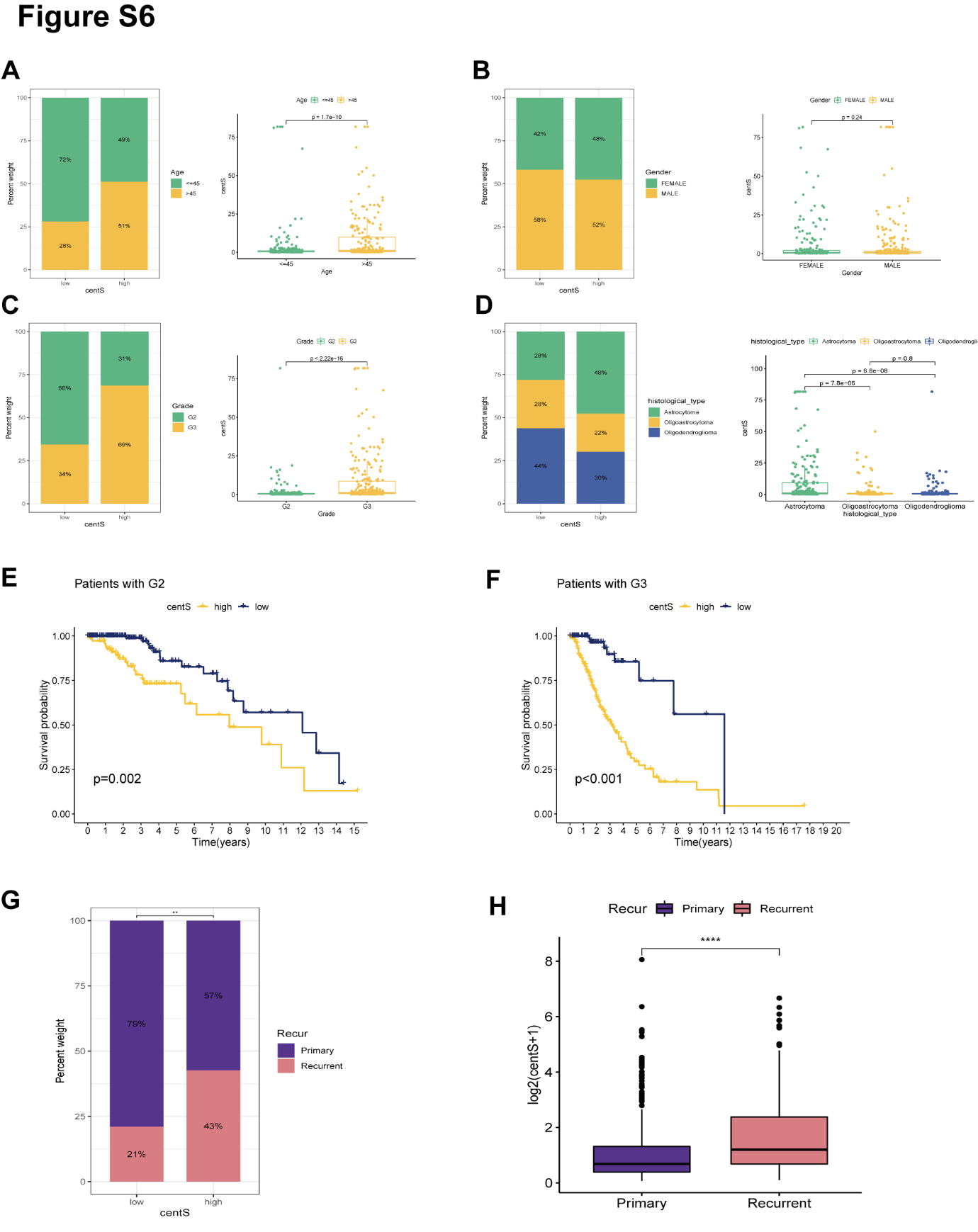


**Supplementary Figure6**. Clinical subgroup analysis based on centS in LGG. (**A–D**) The relationship between four clinical characteristics and centS by ratio distribution and difference comparison: Age (**A**), Gender (**B**), Grade (**C**), histological_type (**D**). (**E-F**) K-M survival analysis based on centS grouping in LGG patients of G2 grade (**E**) and G3 grade(**F**). (**G-H**) The relationship between tumor recurrence and centS by ratio distribution and difference comparison. centS, centrosome score.


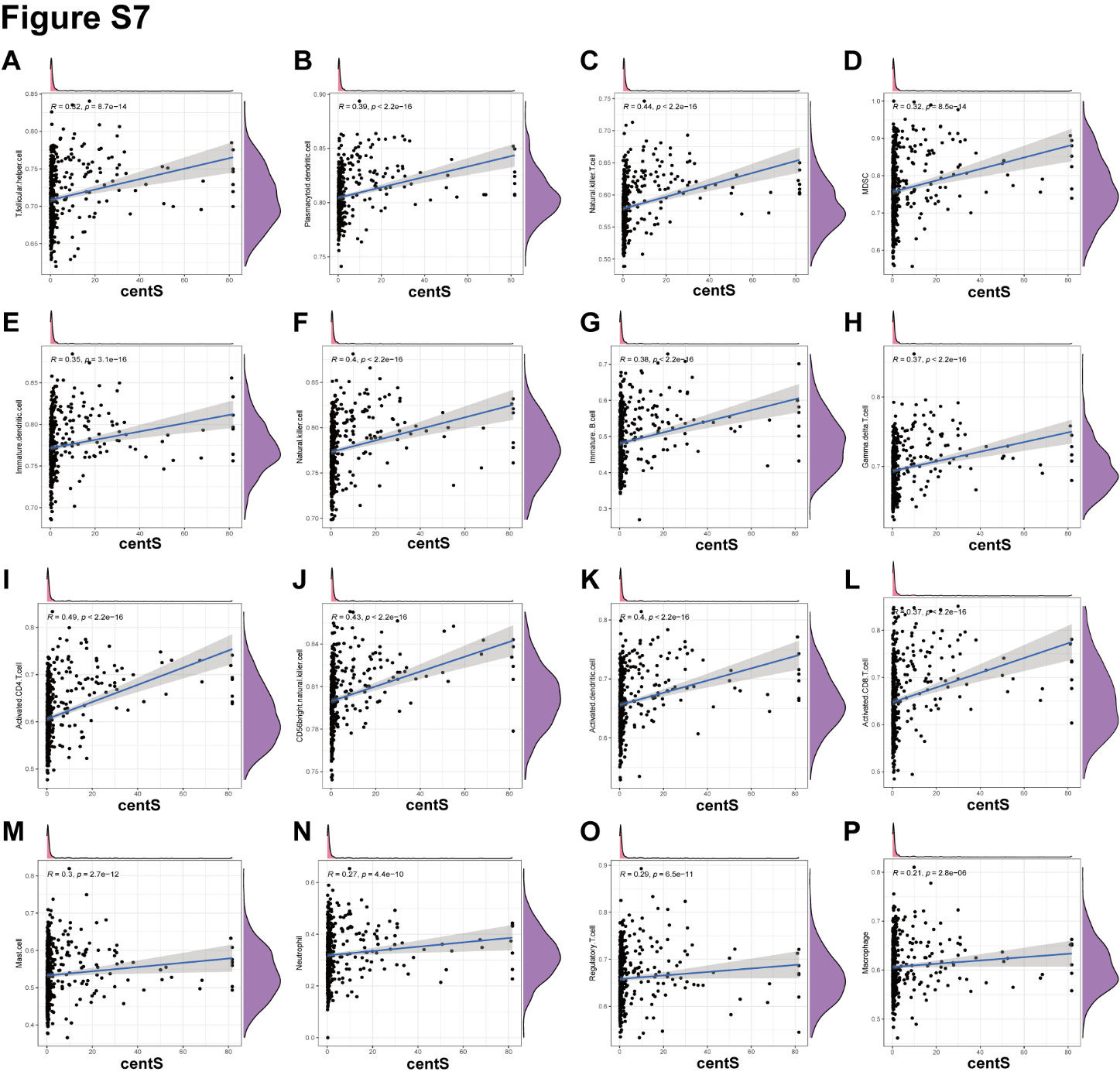


**Supplementary Figure7**. Correlation analysis of centS and positive associated immune cells based on ssGSEA: T.follicular.helper.cell (**A**), Plasmacytoid.dendritic.cell (**B**), Natural.killer.T.cell (**C**), Myeloid-derived suppressor cells (**D**), Immature.dendritic.cell (**E**), Natural.killer.cell (**F**), Immature..B.cell (**G**), Gamma.delta.T.cell (**H**), Activated.CD4.T.cell (**I**), CD56bright.natural.killer.cell (**J**), Activated.dendritic.cell (**K**), Activated.CD8.T.cell (**L**), Mast.cell (**M**), Neutrophil (**N**), Regulatory.T.cell (**O**) and Macrophage (**P**). centS, centrosome score.

## Supplementary Tables

**Supplementary Table S1** **|** Information of primer sequence in qRT-PCR.

| Gene Symbol | NCBI ID | Sequence（5' to 3'） | |
| --- | --- | --- | --- |
| CEP135 | 9662 | F | AACTGAGAGCCTTCGGCAAT |
|  |  | R | ACGTGTTGGTCTGAATGTTCTCT |
| SAMD9 | 54809 | F | ACAATACCCATCACTCCCGC |
|  |  | R | TCATAAGCAAGTGGGCCTCC |
| SMC4 | 10051 | F | CTTACTTTGGGAGGGGACGC |
|  |  | R | GTAAAGGGGAGTGGGCTTGT |
| WEE1 | 7465 | F | GCTTGCCCTCACAGTGGTAT |
|  |  | R | GCACTTGTGGTATCCGAGGT |
| IGFBP2 | 3485 | F | ATCTCCACCATGCGCCTTC |
|  |  | R | TGTTTGAGGTTGTACAGGCCAT |
| DPEP1 | 1800 | F | CATCTTCAGCCACTCCTCGG |
|  |  | R | TTCACCATCACCAGGCTGTC |
|  |  |  |  |

Notes: F: Forward; R: Reverse.

**Supplementary Table S2** | Results of multivariate Cox regression analysis of the 12 model genes.

| Gene | HR (95%CI low-95%CI high) | p value |
| --- | --- | --- |
| SMC4 | 2.26 (1.93-2.64) | 1.39E-24 |
| WEE1 | 2.18 (1.88-2.53) | 2.73E-25 |
| SAMD9 | 1.95 (1.67-2.29) | 1.01E-16 |
| IGFBP2 | 1.57 (1.45-1.70) | 7.03E-29 |
| MMP9 | 1.21 (1.10-1.33) | 6.77E-05 |
| DPEP1 | 1.36 (1.22-1.52) | 3.54E-08 |
| TGFBI | 1.16 (1.03-1.31) | 1.39E-02 |
| F5 | 0.87 (0.79-0.97) | 1.11E-02 |
| HOXA10 | 1.48 (1.34-1.63) | 2.36E-15 |
| EMP3 | 1.66 (1.52-1.81) | 1.74E-28 |
| TFPI | 1.48 (1.30-1.69) | 4.62E-09 |
| SFRP2 | 0.76 (0.70-0.81) | 3.75E-15 |

**Supplementary Table 3** **|** Results of univariate Cox regression analysis and AUC of the 13 centrosome related genes.

| Gene | HR (95%CI low-95%CI high) | | | | p value | AUC |  |
| --- | --- | --- | --- | --- | --- | --- | --- |
| STIL | 2.24 (1.86-2.71) | | | | 4.67E-17 | 0.80 |  |
| SASS6 | 2.82 (2.02-3.94) | | | | 1.35E-09 | 0.65 |  |
| SPICE1 | 2.21 (1.54-3.18) | | | | 1.82E-05 | 0.62 |  |
| PLK4 | 1.96 (1.63-2.36) | | | | 1.06E-12 | 0.79 |  |
| CEP295 | 1.69 (1.20-2.40) | | | | 0.003044 | 0.64 |  |
| POC5 | 3.58 (2.23-5.77) | | | | 1.50E-07 | 0.66 |  |
| CEP152 | 2.38 (1.90-2.98) | | | | 4.32E-14 | 0.74 |  |
| CENPJ | 1.29 (0.99-1.69) | | | | 0.059286 | 0.53 |  |
| RTTN | 2.23 (1.67-2.96) | | | | 3.89E-08 | 0.64 |  |
| CEP135 | 2.64 (2.18-3.12) | | | | 8.48E-23 | 0.80 |  |
| CEP63 | 1.86 (1.02-3.42) | | | | 0.043996 | 0.62 |  |
| POC1B  CEP120 | 2.12 (1.35-3.33)  2.77 (1.75-4.36) | | | | 0.001155  1.19E-05 | 0.56  0.70 |  |
|  | |  |  |  | | | |

Notes: AUC: Area Under roc Curve.
